# 1,2,3-Triazoles as Biomimetics in Peptide Science

**DOI:** 10.3390/molecules26102937

**Published:** 2021-05-14

**Authors:** Naima Agouram, El Mestafa El Hadrami, Abdeslem Bentama

**Affiliations:** Laboratory of Applied Organic Chemistry, Faculty of Science and Technology, Sidi Mohammed Ben Abdellah University, Immouzer Road, Fez 30050, Morocco; elmestafa.elhadrami@usmba.ac.ma (E.M.E.H.); abdeslem.bentama@usmba.ac.ma (A.B.)

**Keywords:** 1,2,3-triazole, peptidomimetic, CuAAC, amide bond, click chemistry, bioisostere

## Abstract

Natural peptides are an important class of chemical mediators, essential for most vital processes. What limits the potential of the use of peptides as drugs is their low bioavailability and enzymatic degradation in vivo. To overcome this limitation, the development of new molecules mimicking peptides is of great importance for the development of new biologically active molecules. Therefore, replacing the amide bond in a peptide with a heterocyclic bioisostere, such as the 1,2,3-triazole ring, can be considered an effective solution for the synthesis of biologically relevant peptidomimetics. These 1,2,3-triazoles may have an interesting biological activity, because they behave as rigid link units, which can mimic the electronic properties of amide bonds and show bioisosteric effects. Additionally, triazole can be used as a linker moiety to link peptides to other functional groups.

## 1. Introduction

Peptides are ultra-powerful molecules and have become an essential source of new medical strategies. Peptides are found in all tissues and cells in the human body, and given their involvement in most biological processes, they are of great interest as therapeutic drugs.

During the past decade, several drug synthesis studies have identified peptides as an innovative and growing therapeutic area. They have been used as important therapeutic agents in the treatment of different diseases, e.g., cancer, cardiovascular diseases, and diabetes. Additionally, some peptides have been found to exhibit antiviral activity. These antiviral peptides (AVPs) exhibit activity against a broad spectrum of viruses [1,2,3,4,5]. Therefore, recent trials have demonstrated the interesting therapeutic effect of AVPs against SARS-CoV-2 (severe acute respiratory syndrome coronavirus 2), which has become a worldwide pandemic [6]. Another study has demonstrated the efficacy of CIGB-258, an immunoregulatory peptide, in the treatment of COVID-19 (coronavirus disease 2019), improving the clinical status of COVID-19 patients [7]. In addition to the clinical trials for the treatment of COVID-19, the vaccine remains the most promising solution. A newly designed multi-peptide subunit-based epitope vaccine against COVID-19 was studied and showed a good protective efficacy and safety against SARS-CoV-2 infection [8]. 

The use of peptides as drugs is limited, because they have intrinsic weaknesses: variable solubility, low bioavailability, and limited chemical and physical stability [9,10]. Peptides are prone to degradation, and they have a short half-life due to in vivo enzyme degradation (gastrointestinal, plasma, and tissue peptidases) [11]. 

To overcome this limitation, some modifications are made to peptides in order to synthesize new molecules that biologically mimic peptides, such as peptidomimetics, for the development of useful therapeutic agents [12,13].

An effective strategy to guide the optimization of peptidomimetics is the theoretical approach [14] to increase the potential of peptidomimetics as therapeutic products. This approach consists of combining different computational strategies [15,16,17,18,19,20,21], ranging from bioinformatics approaches to predicting the conformational plasticity of peptidomimetics, to molecular simulations to predict the binding affinity of peptidomimetics for their targets.

The use of peptidomimetics is one of the most important methods of drug design and development in medicinal chemistry. They are elements that are designed to mimic a peptide and retain the ability to interact with a biological target and produce the same biological effect, and they are designed to advantageously adjust various properties, such as stability against proteolysis (duration of activity), and circumvent some problems, such as low bioavailability.

An important example of peptide mimicry in medicinal chemistry is the replacement of the amide with a heterocyclic bioisostere such as the 1,2,3-triazole moiety [22,23].

**Figure 1:**
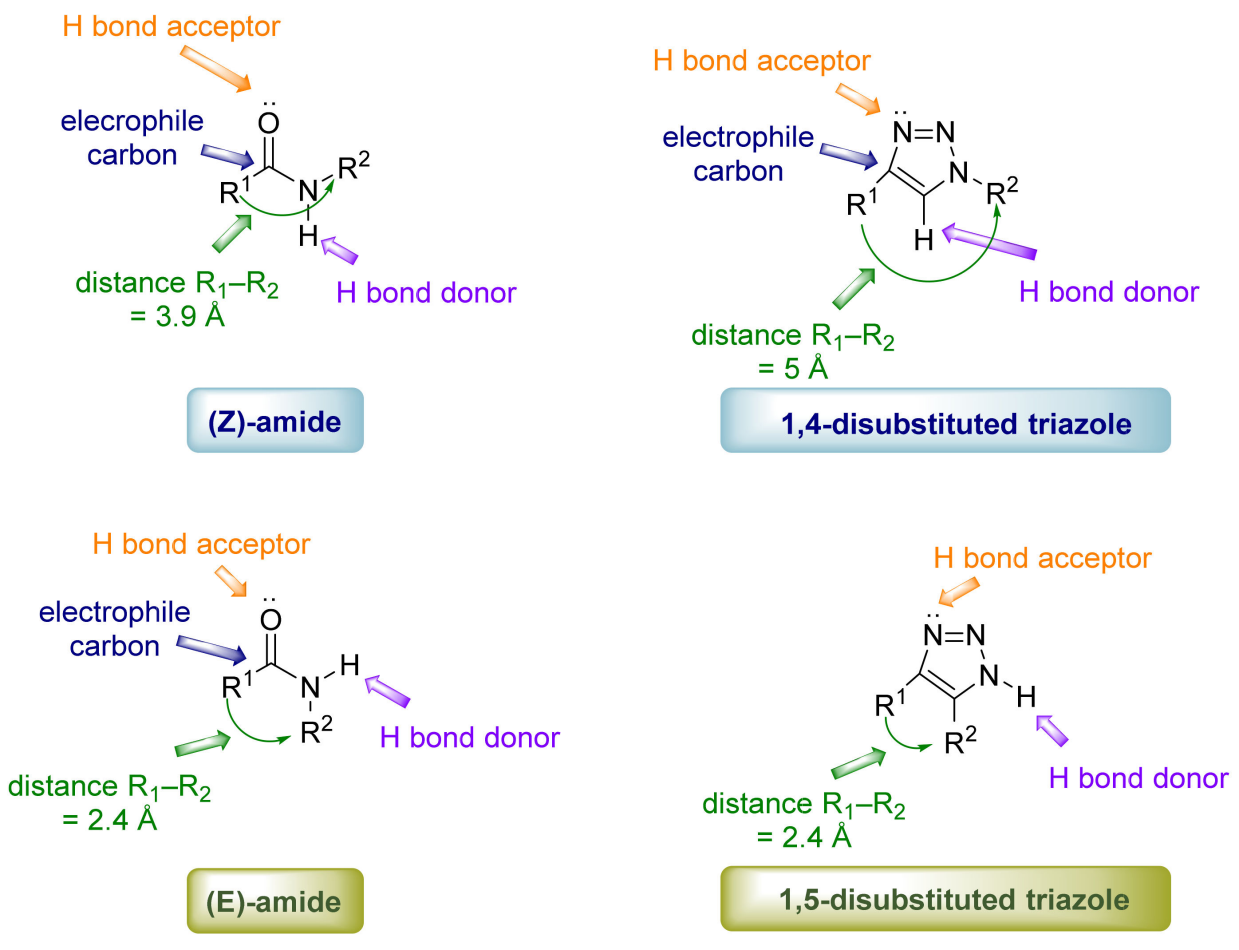
Topological and electronic mimicry of amide units by 1,2,3-triazoles.

The structure of 1,2,3-triazole suggests that this ring may mimic the peptide bond (amide function) in either a *cis* or *trans* configuration. The 1,4-disubstituted triazole demonstrated similarity to the amide bond in the *trans* configuration. The lone pair of nitrogen mimics the carbonyl oxygen of the amide, and the polarity of the C(5)–H bond is also a mimic of the hydrogen bond donor character of the N–H bond, while the C(4) atom is electronically similar to the amide carbonyl carbon (Figure 1) [24]. The dipolar moment of the triazole ring is higher than that of the amide function, and the hydrogen bond donor/acceptor power is more important, thus allowing for the optimization of peptide mimicry. The difference between the 1,4-disubstituted 1,2,3-triazole ring and the *trans*-peptide bond is the distance between the substituents, R^1^ and R^2^ (Figure 1). 

Additionally, certain 1,5-disubstituted 1,2,3-triazoles can mimic the cis-peptide bond, but with a different polarity.

“Click” chemistry, a concept introduced by Sharpless et al. [25] in 2001, is an approach to synthesis that makes use of reliable and rapid reactions. It was first invented to describe efficient and highly selective reactions that bind molecules with a high yield. Among these reactions, the most popular is the Huisgen cycloaddition, which is a 1,3-dipolar cycloaddition between an azide moiety and alkyne function leading to the formation of the triazole cycle. It is a [3+2] cycloaddition that proceeds in a similar manner to Diels–Alder’s reaction.

Triazoles have a number of desirable characteristics in the context of drug discovery. For example, they support acid and basic hydrolysis and withstand conditions of reduction and oxidation, thus indicating their stability. At the same time, this heterocycle has a high dipolar moment (approximately 5D) and is expected to actively participate in hydrogen bonding, as well as in dipole–dipole interactions and π–π stacking. It is also resistant to metabolic degradation.

**Scheme 1:**
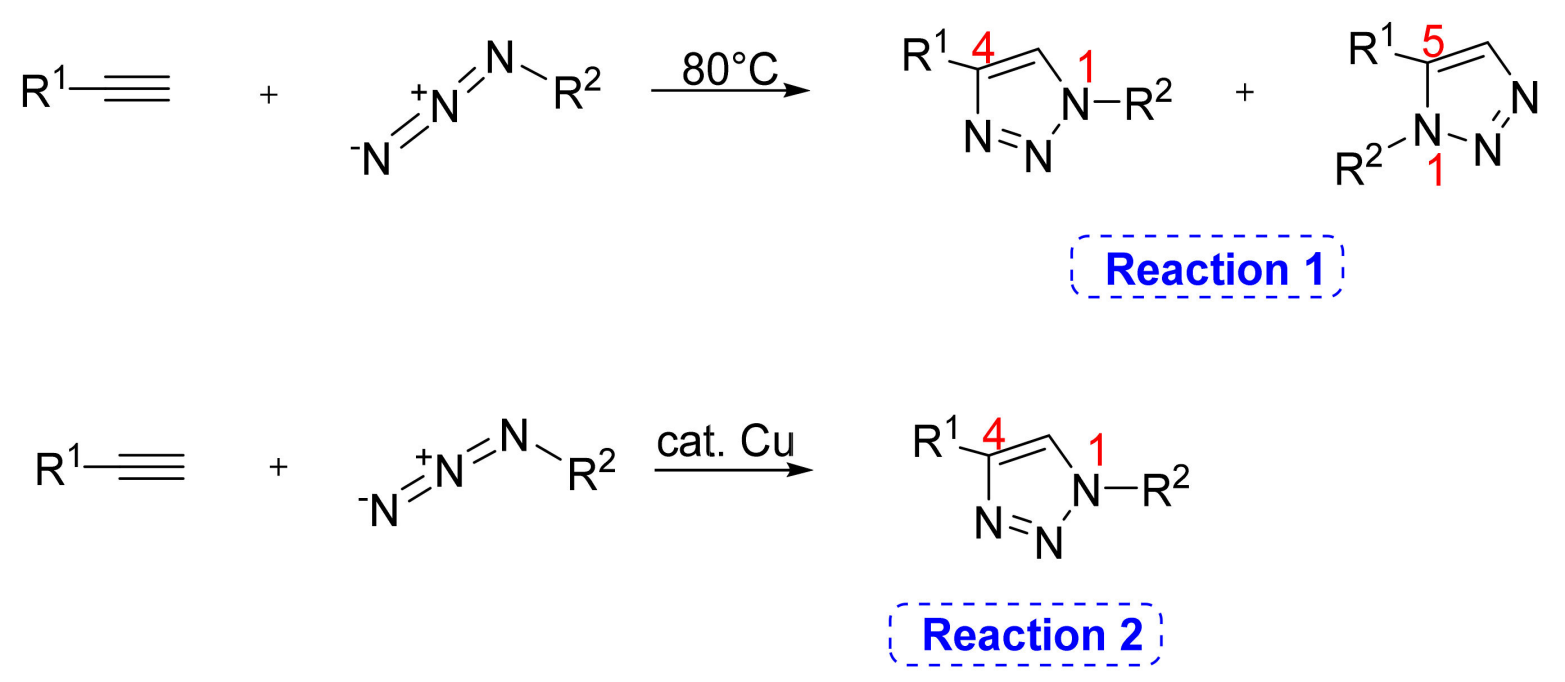
Preparation of triazole: (reaction 1) uncatalyzed Huisgen cycloaddition leading to 1,4₋ and 1,5₋regioisomer; (reaction 2) copper₋catalyzed azide₋alkyne cycloaddition (CuAAC) leading to 1,4₋regioisomer.

In addition, a copper catalyst is used to obtain only the 1,4-regioisomer (1,4-disubstituted-1*H*-1,2,3-triazole) (Scheme 1: reaction 2) in contrast to the uncatalyzed Huisgen reaction (Scheme 1: reaction 1) [26]. There are many benefits of this reaction. In particular, it leads to pure products, requires very simple reaction conditions, uses less harmful solvents, leads to very high yields, does not generate byproducts, and can be applied in many areas.

In general, the incorporation of click chemistry in peptide synthesis [26,27] has greatly advanced peptide science. As a result of the reaction conditions, efficiency, and selectivity of this reaction, it is an effective tool for linking groups together (such as peptide fragments) to ligate smaller fragments for the synthesis of large peptides and for peptide cyclization (side-chain to side-chain or head-to-tail). The introduction of triazoles can mimic and rigidify the conformation of the amide backbone. In addition, the triazole has widely been used to link peptides to various functional groups, such as radioactive molecules or fluorescent compounds, carbohydrates, and other bioactive molecules. This conjugation effectively increases the function of peptides. Moreover, triazoles are considered as a promoter for C–H bond activations and are used as a directing group (DG) allowing the functionalization of primary and secondary C–H bonds of peptides in order to assemble triazolopeptides [28,29,30]. 

## 2. 1,2,3-. Triazole Moiety and Peptide Structure

In the following sections, we describe the incorporation of the 1,2,3-triazole moiety into peptide structures and the study of compounds containing the triazole motif.

### 2.1. Acyclic Analogues

#### 2.1.1. Peptidotriazole

**Figure 2:**
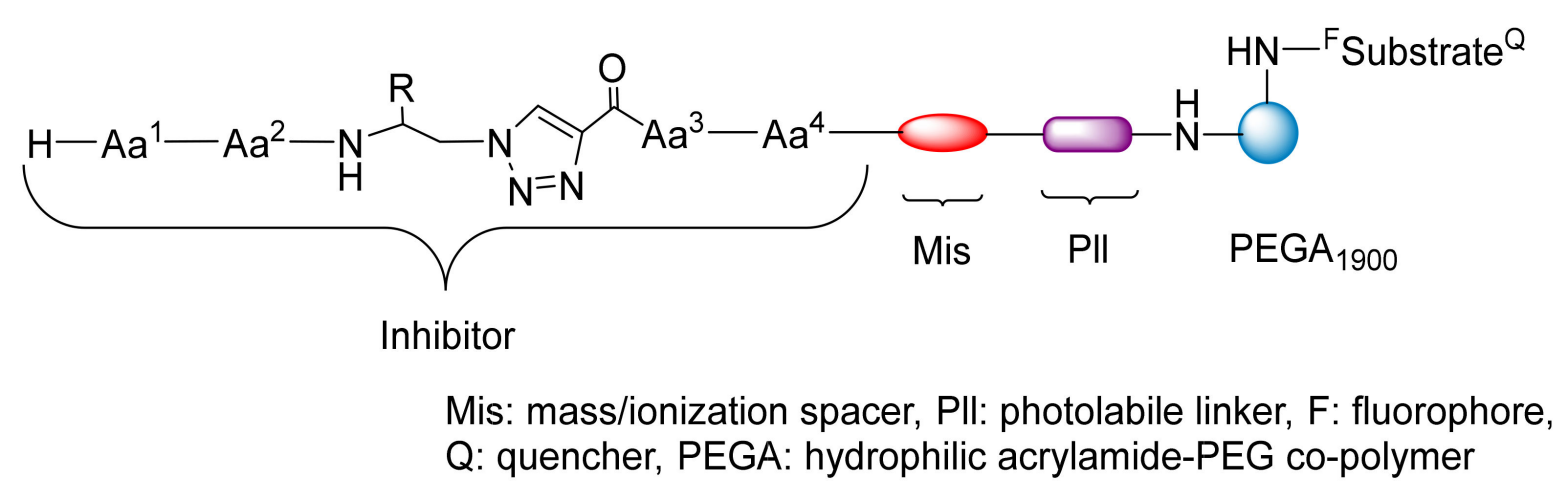
Structure of the peptidotriazole library.

The Meldal group prepared a huge library of compounds (over 400,000) through the split and mix approach [31] and incorporated them into the hydrophilic acrylamide–PEG co-polymer (PEGA) resin. Figure 2 shows the basic structure of the target compounds.

First, the amine of the resin was protected by two different protective groups (Alloc and Fmoc), thereby facilitating the coupling of the substrate to one amine and the introduction of the inhibitor to the other amine. This has been functionalized with a photolabile linker group (Pll), followed by a peptide chain (Mis) to increase the sensitivity of the structure to mass spectrometry analyses in MALDI mode. Two amino acids were then coupled to the N-terminal end of the chain, the latter being coupled to the amide of propiolic acid. Subsequently, copper-catalyzed cycloadditions were made quantitatively in a randomized fashion using five Boc/Fmoc-β-aminoazides, leading to peptide analogs containing 1,4-substituted 1,2,3-triazole ring. By a combinatorial approach, triazole is flanked on both sides by two amino acids, allowing for the formation of a combinatorial library of protease inhibitors of the *Leishmania mexicana* parasite. 

The copper (I)-catalyzed 1,3-dipolar cycloaddition (CuAAC) combined with solid-phase peptide synthesis (SPPS) resulted in a library of protease-bound peptidomimetics with resistance to hydrolysis, thanks to the structural mimetic, 1,2,3-triazole, within the peptide backbone. 

**Figure 3:**
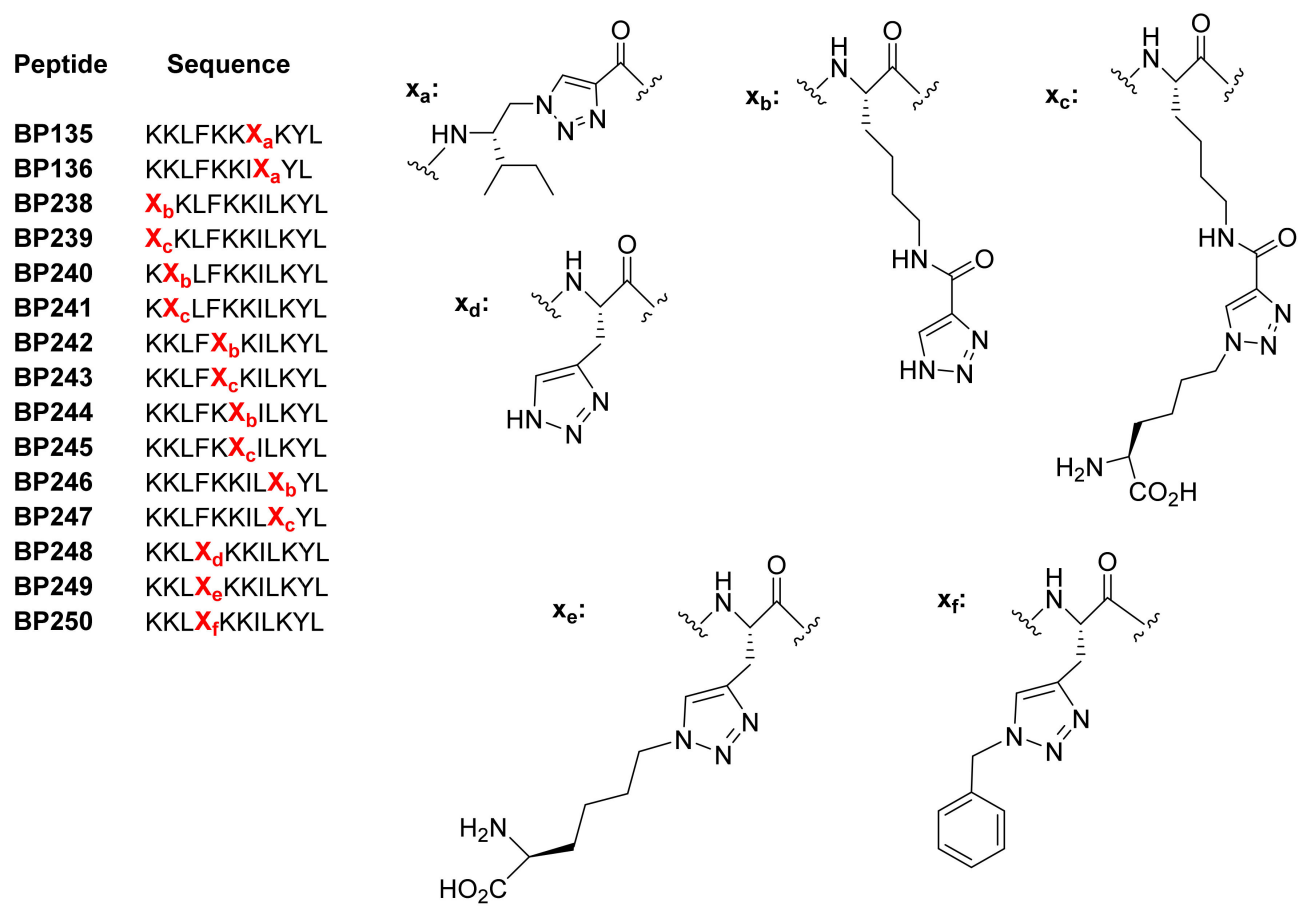
Structure of BP100 peptide sequence analogues.

Another example of peptide bond substitution by 1,4-disubstituted triazole has been reported by Guell et al. in the synthesis of peptidotriazoles with antimicrobial activity [32]. Indeed, analogues of the antimicrobial peptide BP100 (Lys-Lys-Leu-Phe-Lys-Lys-Ile-Leu-Lys-Tyr-Leu-NH_2_) were prepared by introducing the triazole ring on the side chain of Lys or Phe (Figure 3). This allowed for the identification of active sequences against the bacteria *Xanthomonas axonopodis pv. vesicatoria, Erwinia amylovora*, and *Pseudomonas syringae pv. syringae* and against the fungus, *Fusarium oxysporum*, with a low hemolytic activity, high stability in protease digestion, and no phytotoxicity.

**Figure 4:**
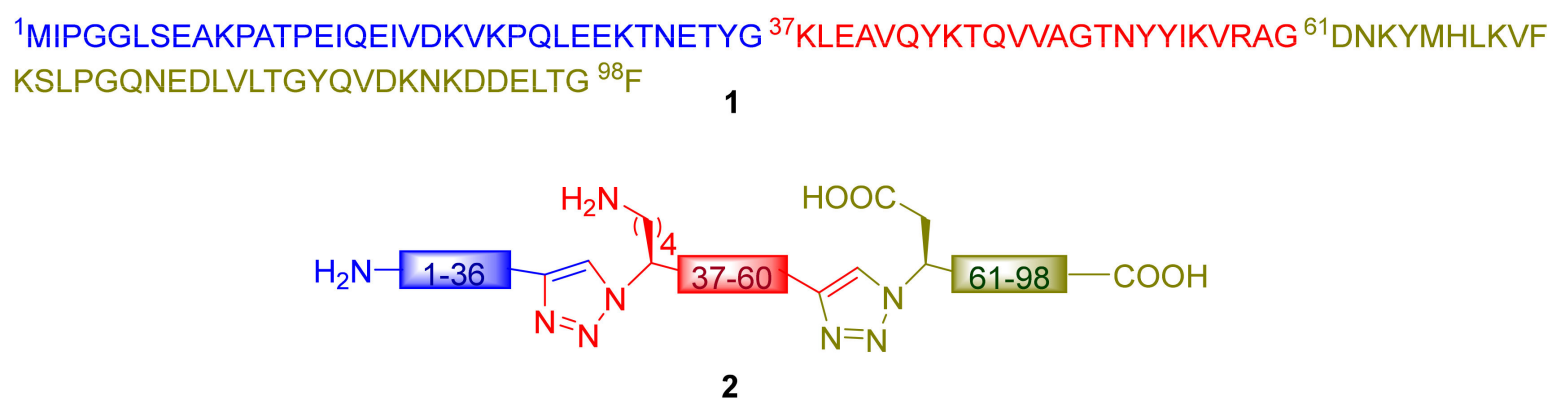
Sequence of cystatin A (**1**) and structure of the related triazole containing peptidomimetic (**2**).

Delmas et al. [33] prepared cystatin A containing two triazole groups from three peptide fragments of the protein (Figure 4). This cystatin A, analogue **2**, has inhibitory activity in the cathepsin invasion of breast cancer cells.

In natural proteins, the vast majority of peptide bonds are in a *trans* conformation, however *cis* conformation peptide bonds sometimes occur. The *cis* configuration is observed in the peptide bonds containing proline, because the free energy difference between *cis* and *trans* isomers is much smaller. *Cis* conformation peptide bonds can have an impact on protein structure and function. *Cis*-prolyl peptide bond surrogates were evaluated. 

**Figure 5:**
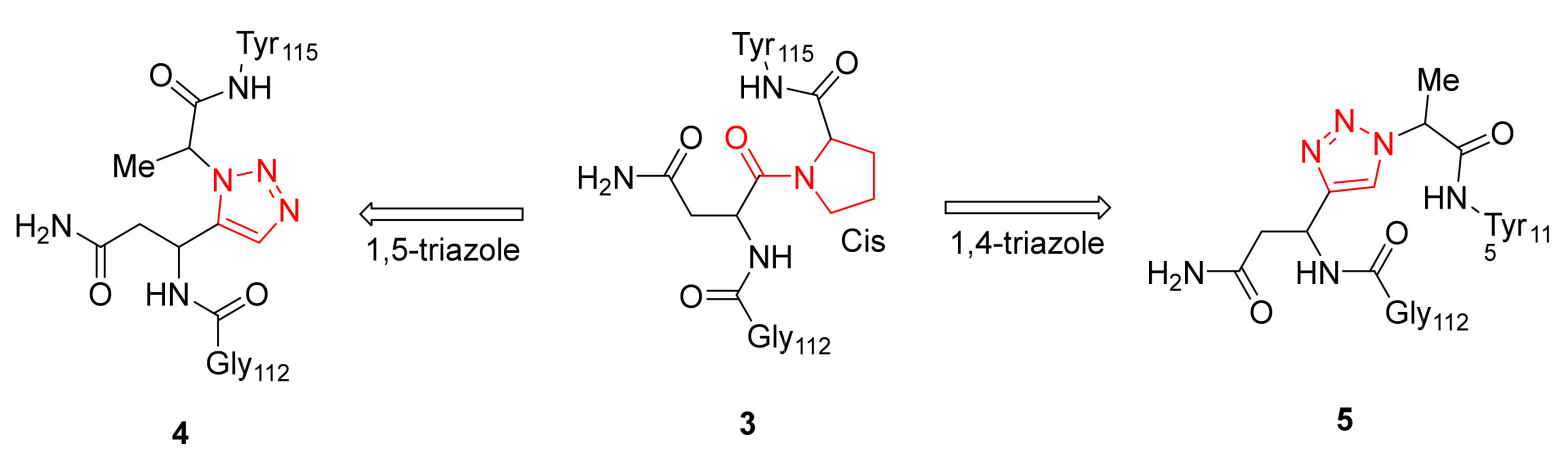
Substitution of the *cis*-prolyl peptide bond with 1,4- and 1,5-disubstituted triazole rings.

Raines et al. [34] examined the possibility of introducing 1,4-disubstituted triazole or 1,5-disubstituted triazole, instead of a *cis*-prolyl peptide bond (Figure 5). Compounds with a 1,4-disubstituted triazole moiety (**5**) exhibit a complete enzymatic activity indicative of the maintenance of the native structure, and compounds with a 1,5-isomer (**4**) exhibit a similar conformational stability to that of *cis*-Xaa-Pro segments **3**, unlike **5**.

Dipeptide mimetics are prepared from azide and acetylene derivatives using either the CuAAC or RuAAC methodology. Two 1,4-triazole pseudodipeptides and their 1,5-regioisomer were then incorporated in a turn region of bovine pancreatic ribonuclease (RNase A). All resultant peptidomimetics retained a complete catalytic activity, with the CD (circular dichroism) spectra compatible with maintaining the secondary structure associated with native proteins. Therefore, 1,5-substituted triazole peptidomimetics have the same temperatures of thermal denaturation (*T_m_*) as native proteins; however, 1,4-substituted triazole peptidomimetics have a lower *T_m_* than the native protein. This shows that 1,5-substituted triazole dipeptides are excellent mimics of *cis*-prolyl bonds in the wild-type protein.

**Scheme 2:**
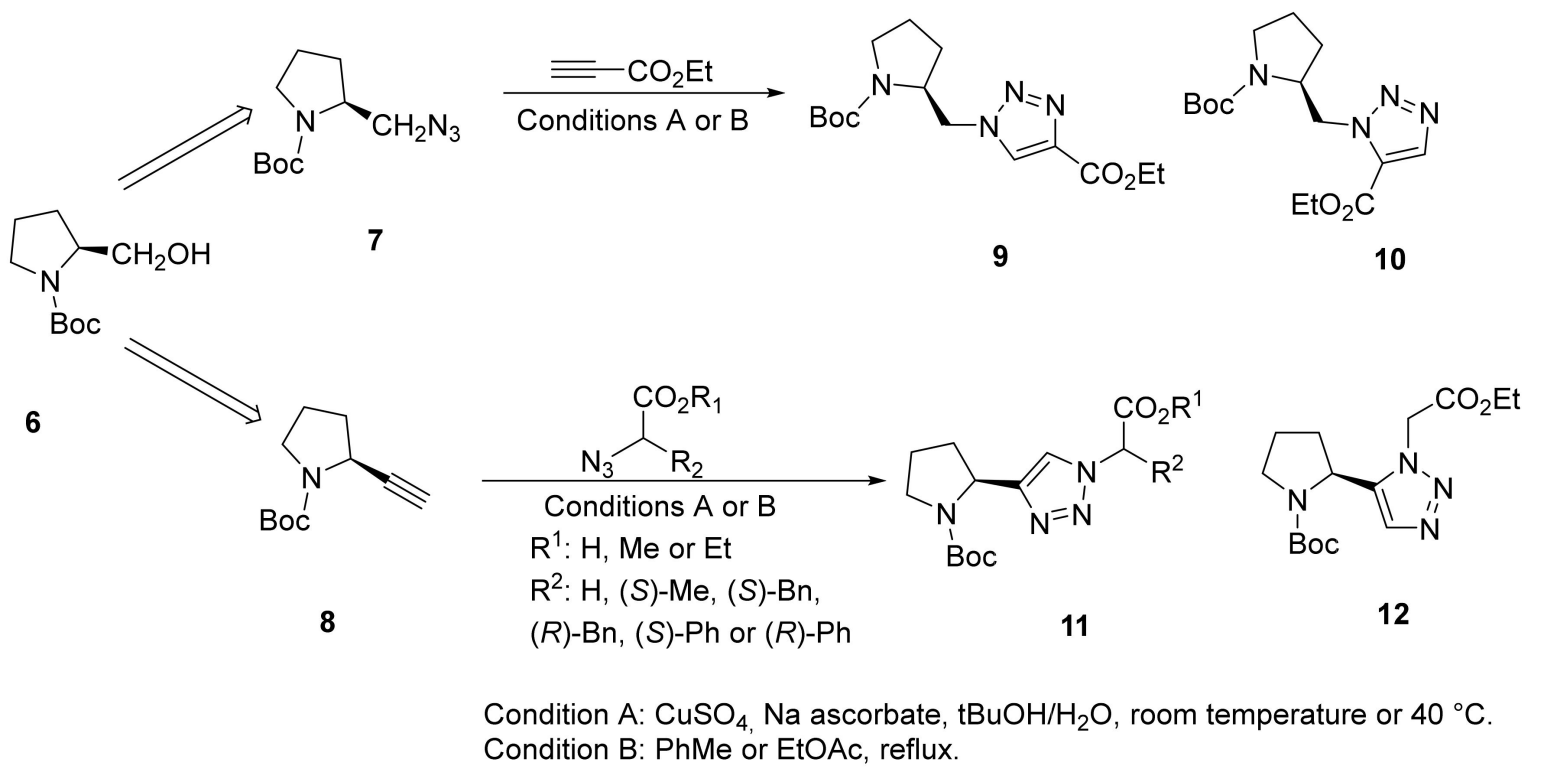
Synthesis of the triazole Pro-Gly dipeptide.

The possibility of controlling the *cis*/*trans* ratio of the amide of proline residues is of considerable interest (e.g., in activating and disabling receptors). In this context, Paul et al. [35] studied the effect of the substitution of an amide bond in a Pro-Gly dipeptide with 1,4- or 1,5-triazole in an attempt to increase the population of *cis* or *trans* conformations. The necessary acetylene and azide (**7** and **8**) (Scheme 2) were synthesized from the protected N-Boc prolinol **6**, and the formation of triazole was achieved either by the copper-catalyzed cycloaddition of Huisgen to give 1,4-substituted triazoles **9** and **11** (36–92%) or by the thermal cycloaddition of Huisgen to produce mixtures of 1,4- and 1,5-disubstituted triazoles in a ratio of about 3:1 (78 and 90% of the combined yield). In most cases, the copper(I)-catalyzed alkyne–azide cycloaddition (CuAAC) was performed using the standard mixture of CuSO_4_/sodium ascorbate in tBuOH/water (in some cases, water was replaced by methanol for solubility reasons) and at room temperature or 40 °C. For triazoles **11** (R^2^ = Ph), it was necessary to perform the cycloaddition at room temperature and to stop the reaction after 20 h to avoid racemization in the phenyl glycine fragment.

**Figure 6:**
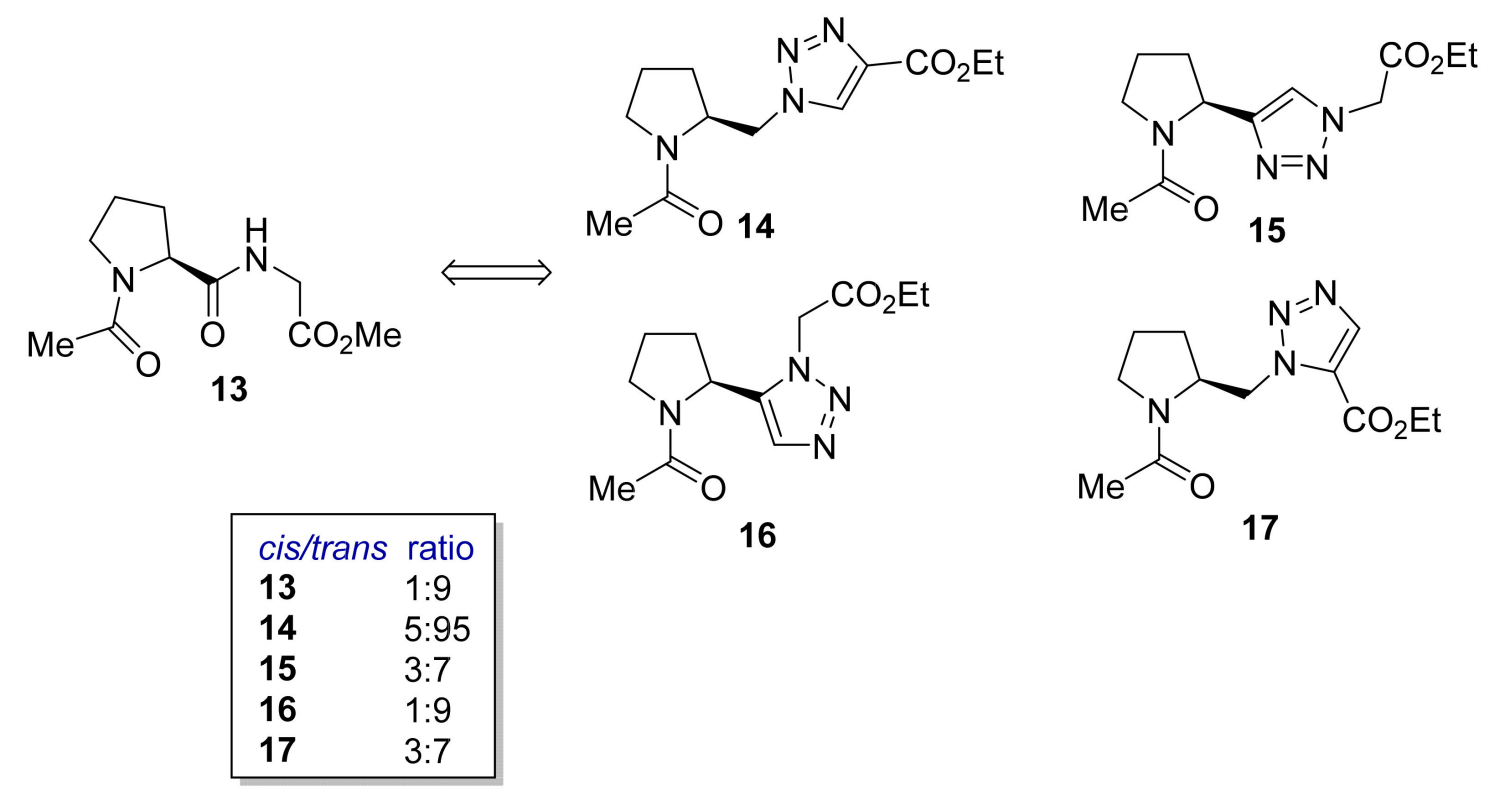
Peptidomimetics of the *cis*-amide bond.

The conformational properties of the synthesized dipeptide mimetics (**14**–**17**) were examined by NMR spectroscopy, and the results were compared with those of the natural dipeptide derivative, *N*-acetylprolyl-glycine methyl ester **13** (Figure 6). Two of the synthesized peptidomimetics (**15** and **17**) showed an increased preference for *cis* conformation (a *cis*/*trans* ratio 3:7) compared to the natural dipeptide derivative **13**. The reason for the increased *cis* conformation in peptidomimetics **15** and **17** has not been determined, but the authors believe that the effect may be due to the dipolar interactions (attraction and repulsion) between the triazole fragment and the carbonyl group in the N-acetyl substituent.

**Scheme 3:**
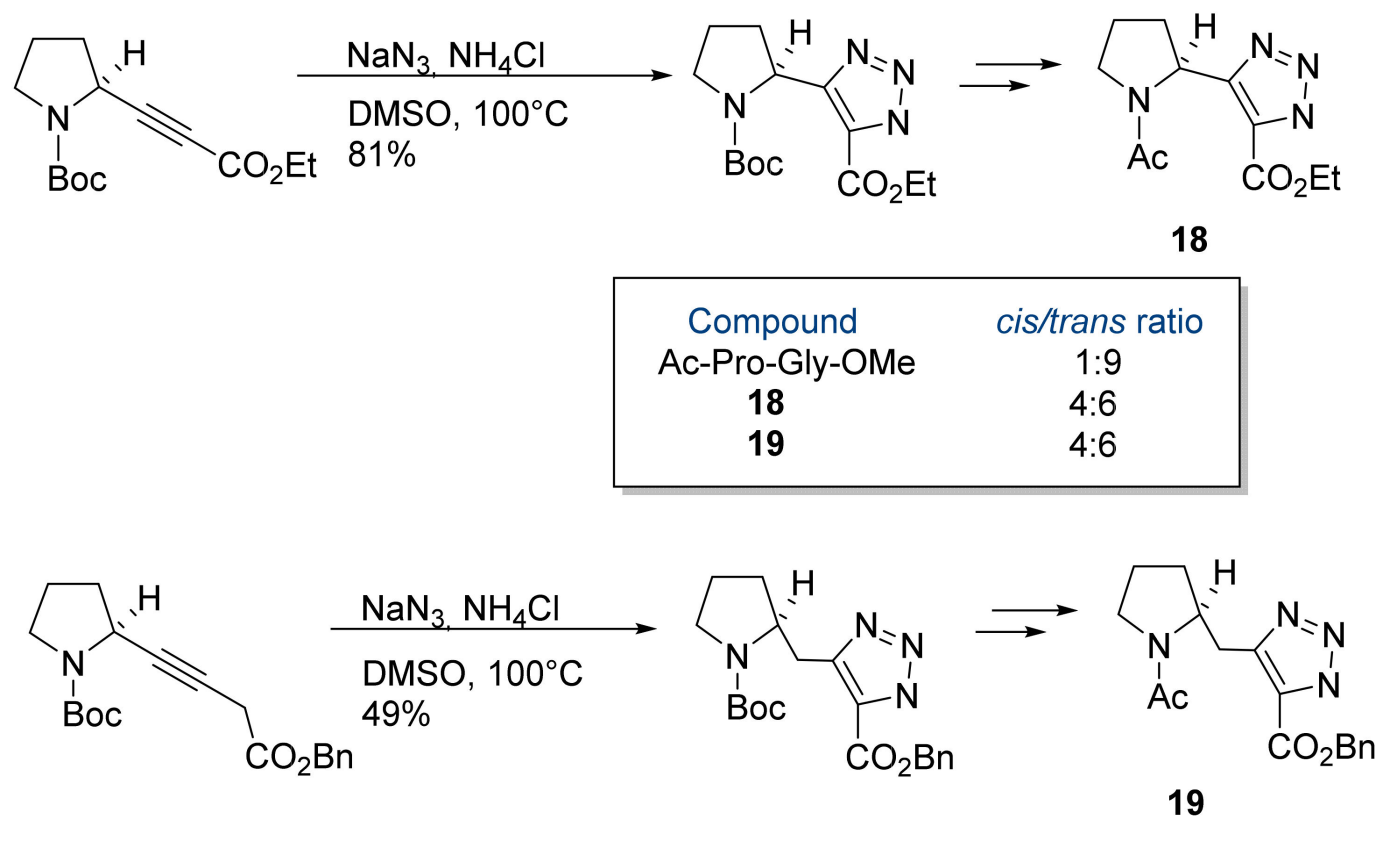
Synthesis of 4,5-substituted triazole peptidomimetics.

More recently, the same authors have published a new approach incorporating the 4,5-disubstituted triazoles [36]. The target molecules were synthesized by Huisgen thermal cycloaddition of hydrazoic acid (formed in situ) and disubstituted alkyne (Scheme 3) or a rearrangement reaction (Scheme 3). A series of triazolic peptidomimetics were synthesized in this way, two of which (**18** and **19**) show an increase in *cis*-prolyl geometry (*cis*/*trans* ration 4:6), compared to the natural dipeptide derivative Ac-Pro-Gly-OMe.

The bioisosteric replacement also enables regulation of the receptor subtype of the peptide. [Y]^6^-Angiotensin II amide bonds were replaced by 1,4-disubstituted 1,2,3-triazole at different positions [37]. Two synthesized peptidomimetics showed increased activity of the AT_2_R/AT_1_R subtype. All [Y]^6^-Angiotensin II derivatives improved the proteolytic stability and retained neurotrophic effects.

#### 2.1.2. β-Turn Mimetics

The interest in preparing compounds containing triazole and peptide fragments stems from the fact that it leads to synthetic peptide analogues with disrupted secondary structures. 

**Figure 7:**
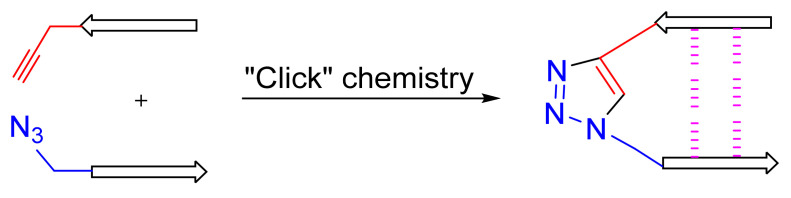
Formation of turns by cycloaddition using “click” chemistry.

Therefore, the CuAAC between two peptide-derivative strands with terminal azide and alkyne functions as an effective synthesis of triazole-based β-turn mimics (Figure 7) [38]. The β-turns are important secondary structures of polypeptides, with α-helices and β-sheets.

Molecular modeling indicates that the propensity to form the intramolecular amide–amide hydrogen bond depends on the spacer length that connects the two amides to the triazole ring. 

**Scheme 4:**
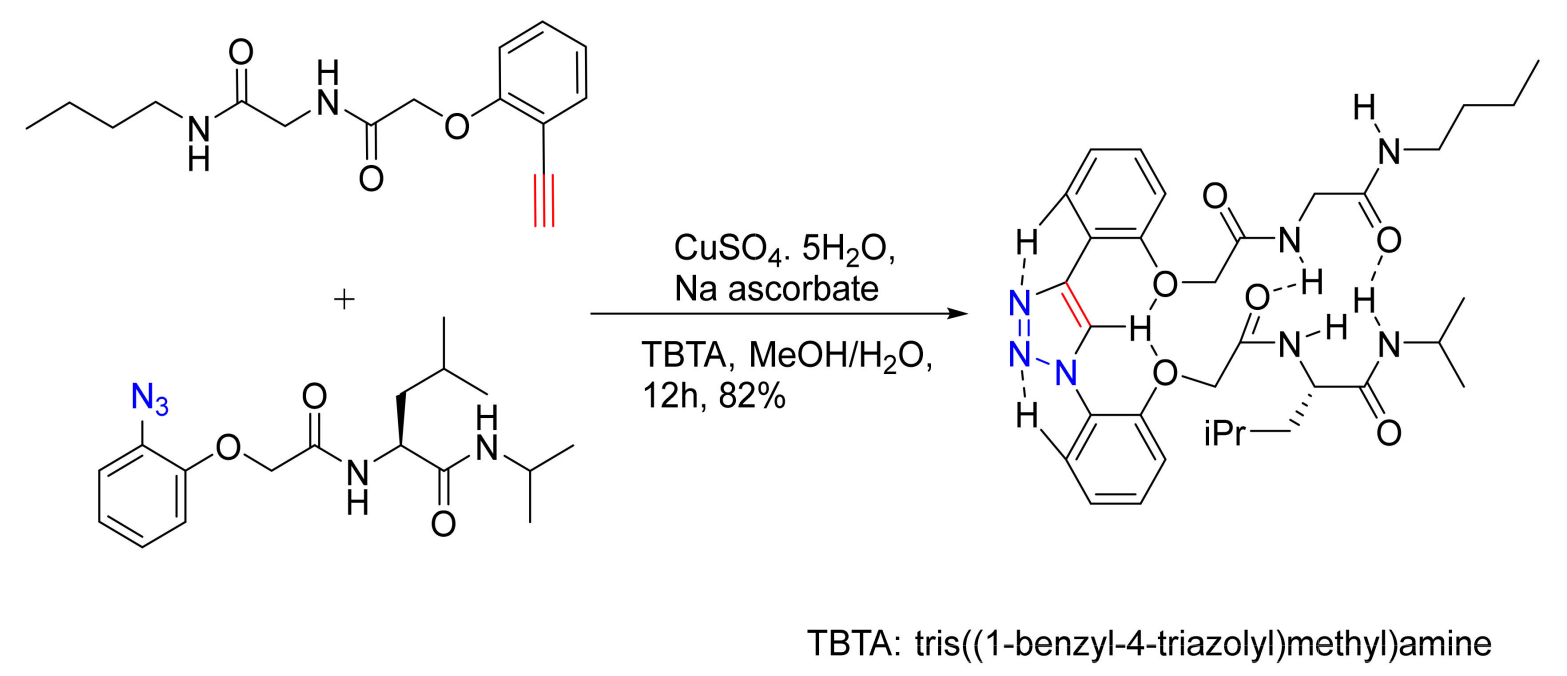
Convergent synthesis of peptide mimetics adopting a β-helix conformation.

Similarly, a 1,4-diphenyl-1,2,3-triazole backbone was synthesized by a convergent approach to rigidify a beta helical conformation (Scheme 4). The X-ray crystallography and 1H NMR results (1D and 2D) revealed that this triazolic derivative folds a U-shaped conformation via the N−H···O intramolecular hydrogen bond, and C−H···O is formed between the triazole C-5 H atom and the two ether O atoms [39].

The fibrillation of amyloid proteins and peptides Aβ [40] is responsible for several neurodegenerative diseases, such as Alzheimer’s and Parkinson’s disease. Inhibitors that prevent amyloid aggregation have recently been studied [41], including the synthesis of β-turn mime conjugates by replacing two amino acids in the turn region of peptide Aβ by the triazole moiety. The synthesis of the triazole aromatic β-turn mime was carried out under the conditions of CuAAC between Fmoc-protected 3-ethynyl aniline and 3-azidobenzoic acid. Through π–π stacking interactions, the β-turn mime can self-assemble. The β-turns inhibit the aggregation of amyloid-β peptides. 

#### 2.1.3. Triazolamer

**Figure 8:**
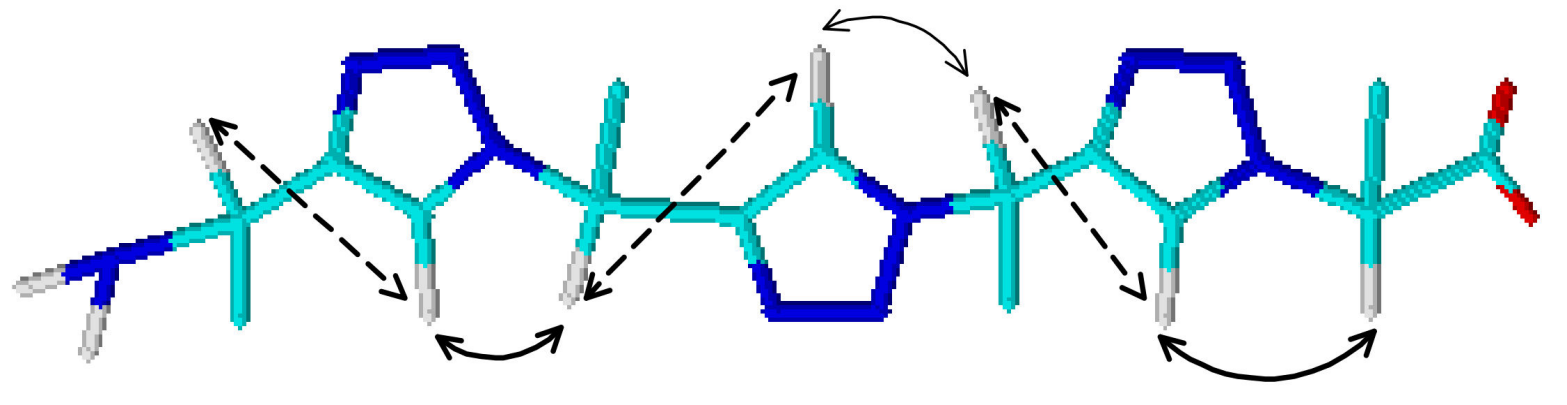
Predominant zig-zag conformation of the triazolamer revealed by the ROESY experiments. The solid and dashed lines indicate strong and weak NOE correlations, respectively. Atom colors: light blue for carbon, dark blue for nitrogen, and red for oxygen.

The Arora group has published reports describing the synthesis of triazole-based oligomers, in which more than four monomer units are linked by a series of triazole rings [42,43,44]. These triazolamers are distinct from peptidotriazole constructions by the fact that the oligomer backbone does not contain any amide bond. The structural evaluation of these oligomers indicated that they fold in discrete “zig-zag” conformations, with an adjacent triazole nucleus turned in opposite directions (Figure 8) [42].

The triazolamer mimics the peptide (strand β), with similar axial distances between the lateral chains i, i+1, and i+2. The overall chirality was retained in relation to similar α-peptide sequences.

The initial synthesis of triazolamers started with standard amino acids and iterative reaction sequences involving the conversion of amine to azide, followed by a CuAAC reaction with the protected amino acid alkyne, deprotection of the amine, and finally, the process was repeated to obtain triazolamers with the desired length.

**Scheme 5:**
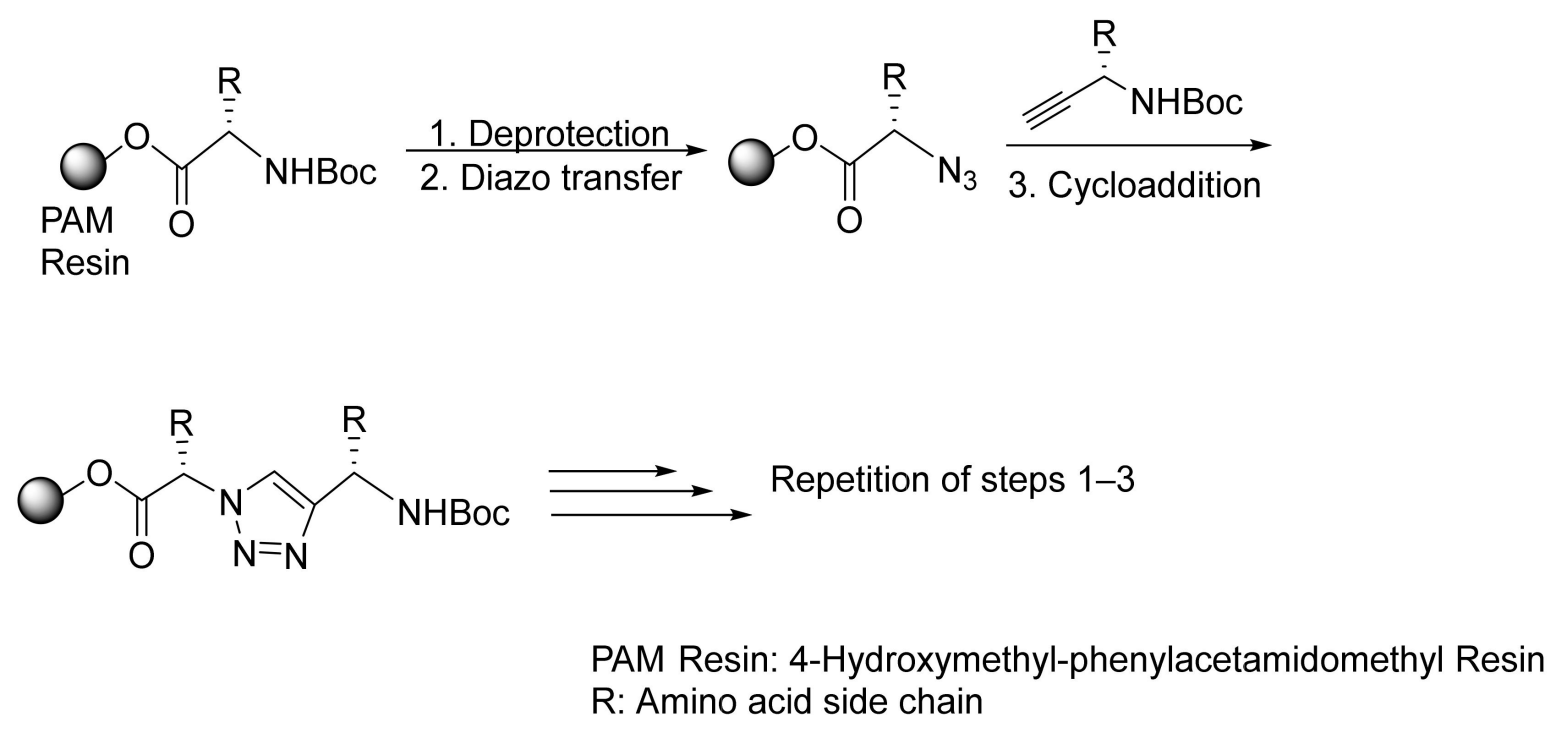
Solid phase synthesis of triazolamers.

This technique leads to low overall yields (6–12%), which is probably due to the instability of the amino acid-derived azide, purification conditions, and inefficiency of the diazo transfer reaction. A better strategy was proposed by synthesizing the three steps in situ in the solid phase, with Zn (II) as a diazo transfer catalyst, followed by the addition of CuSO_4_ to obtain a CuAAC reaction using microwave radiation (Scheme 5) [43]. The final triazolamers were produced with yields above 90%.

**Figure 9:**
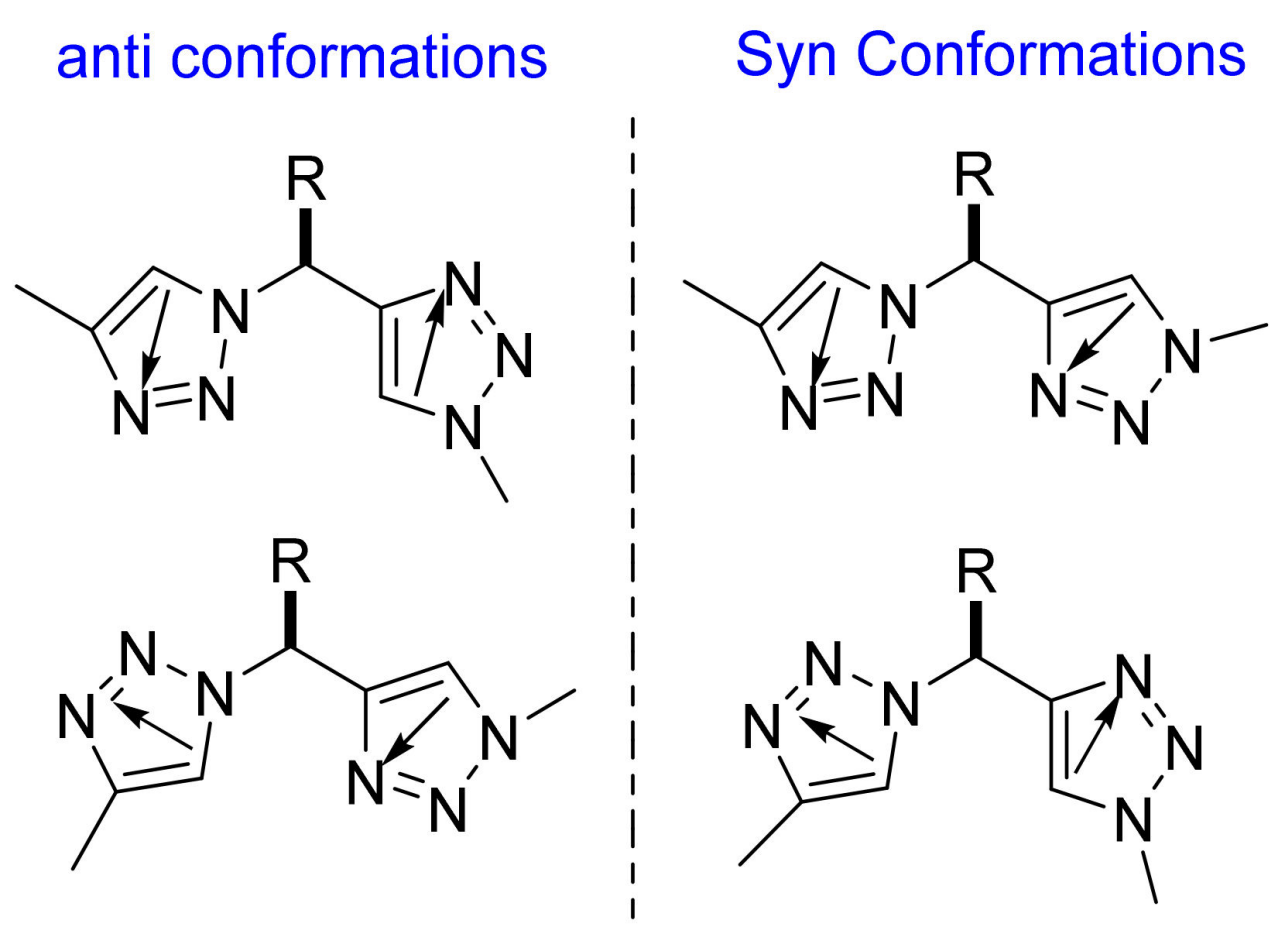
The possible anti-conformations and syn-conformations adopted by the triazole dimer.

An analysis of triazolamer trimer conformations by 1H NMR suggests that they adopt an anti-conformation, which is similar to the β-strand [42]. The Arora group began its studies by calculating the conformation preferences of triazole dimer (Figure 9), which can adopt two anti-conformations and two syn-conformations. Syn- and anti-conformations are defined by the relative directions of the base of the dipoles in the adjacent nucleus. Both molecular mechanics and ab initio studies predict that anti-conformations are more stable than syn-conformations (4 kcal/mol difference).

Proteases are known to link their substrates in a β-strand conformation; therefore, a small library of triazolamers has been tested as HIV-1 protease inhibitors. While synthesized inhibitors were designed exclusively on the basis of possible hydrophobic interactions, several effectively inhibited HIV-1 proteases, with the best compound having an IC_50_ of 25 ± 7 µM [44].

Hughes et al. reported the synthesis of racemic triazolamers using precursor intermediators containing both azide and TMS-protected acetylene. Chiral-substituted homopropargyl-β3 alcohols and chiral-substituted trimethylsilylhomopropargyl-β3 azides were produced from natural l-amino acids. The triazolic chiral oligomers were then prepared using the CuAAC between alkynes and azides [45].

Recently, a new class of peptidotriazolamers has been synthesized [46]. They are peptidomimetics with both the characteristics of triazolamers and peptides, containing 1,4-disubstituted 1*H*-1,2,3-triazoles in alternation with amide bonds. The strategy used for the synthesis of peptidotrizolamer is the CuAAC between chiral propargylamines and chiral α-azido acids. The heptamers, Boc-Ala-Val-Ψ[4Tz]Phe-LeuΨ[4Tz]Phe-LeuΨ[4Tz]Val-OAll, homochiral, heterochiral, and a Gly-substituted derivative adopt a compact folded conformation. In addition, similar oligomers, Boc-Ala-(AlaΨ[4Tz]Ala)6-OAll and Boc-Ala-(d-AlaΨ[4Tz]Ala)6-OAll, predict a well-defined secondary structure, a twisted “S” for the heterochiral compound, and a helix for the homochiral compound in DMSO. 

#### 2.1.4. Coiled Coil Triazoles

The work of Ghadiri’s group demonstrated that triazole units can be used to replace dipeptide sequences within a multi-spiral α-helix, called a “coiled coil” [47].

**Figure 10:**
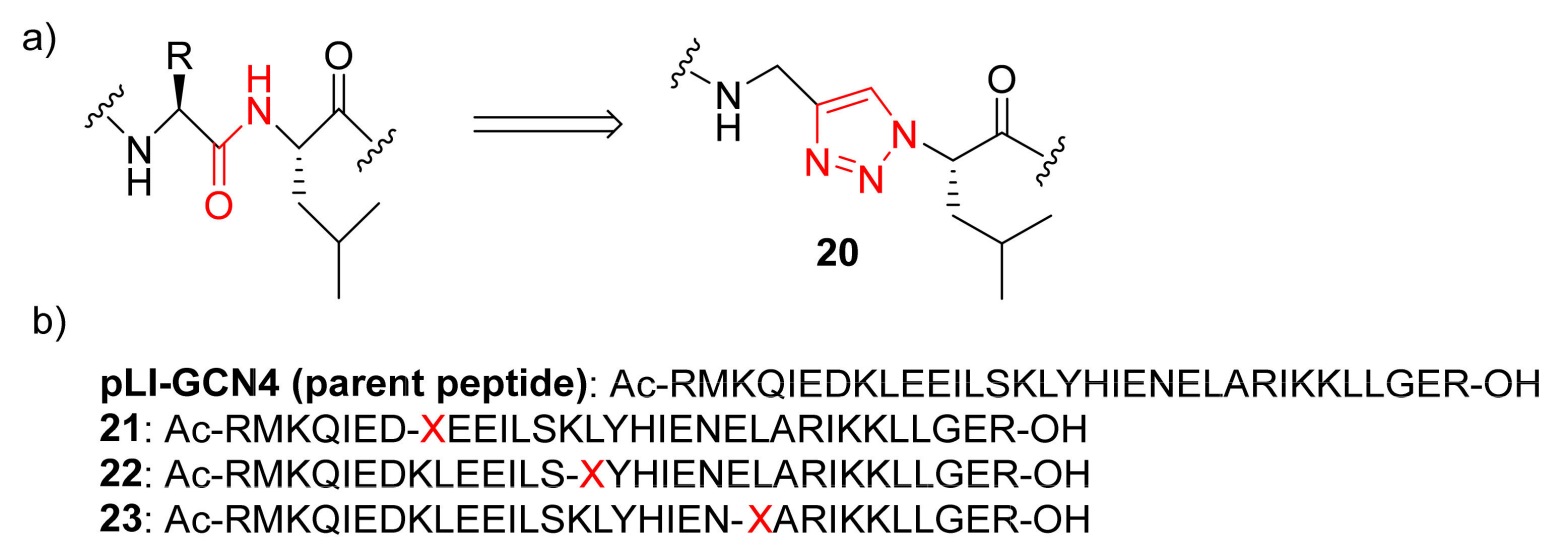
(**a**) Exchange of the dipeptide fragment with the triazole dipeptide mimetic; (**b**) replacement of the native amino acids, pLI-GCN4 K_8_L_9_ (**21**), K_15_L_16_ (**22**), and E_22_L_23_ (**23**), with the peptidomimetic **20**.

Three peptidomimetics were synthesized, with the circular dichroism (CD) characteristic of α-helical structures. Thermal denaturation experiments revealed significant differences between the three peptides, with the only similarity in the temperature of thermal denaturation of peptidomimetic **23** with the parent peptide, pL-GCN4 (Figure 10). This suggests that peptidomimetics **21**–**23** retain a large part of the native α-helix structure, but that the thermodynamic stability of the peptidomimetic is highly dependent on the site of triazole substitution.

The X-ray crystal structure study of peptidomimetics **21**–**23** (at a resolution of 2.2 Å) revealed that all three adopted the same structure observed for the parent peptide, pLI-GCN4.

On the other hand, another study [48] has shown that the position of the triazole insertion in the peptide sequences, as well as the types of the two amino acids surrounding the triazole, can influence the stability of the peptide structure, be responsible for the alteration of the helical secondary structure, and suppress antimicrobial activity.

#### 2.1.5. Amino Acid Mimics

Given the interest in and biological activity of triazoles, amino acids, and glycosidic compounds, and their wide use in different fields, glycosylated triazole amino acids based on l-serine have been synthesized [49]. l-serine, a non-essential amino acid particularly important for the proper functioning of the brain and central nervous system [50,51], was coupled with propargylamine and 4-ethynylaniline, followed by a cycloaddition with glycosylazides, using a catalytic 1,3-dipolar cycloaddition reaction to form 1,2,3-triazole amino acids glycosylated by d-glucose, d-galactose, and d-ribose.

**Scheme 6:**
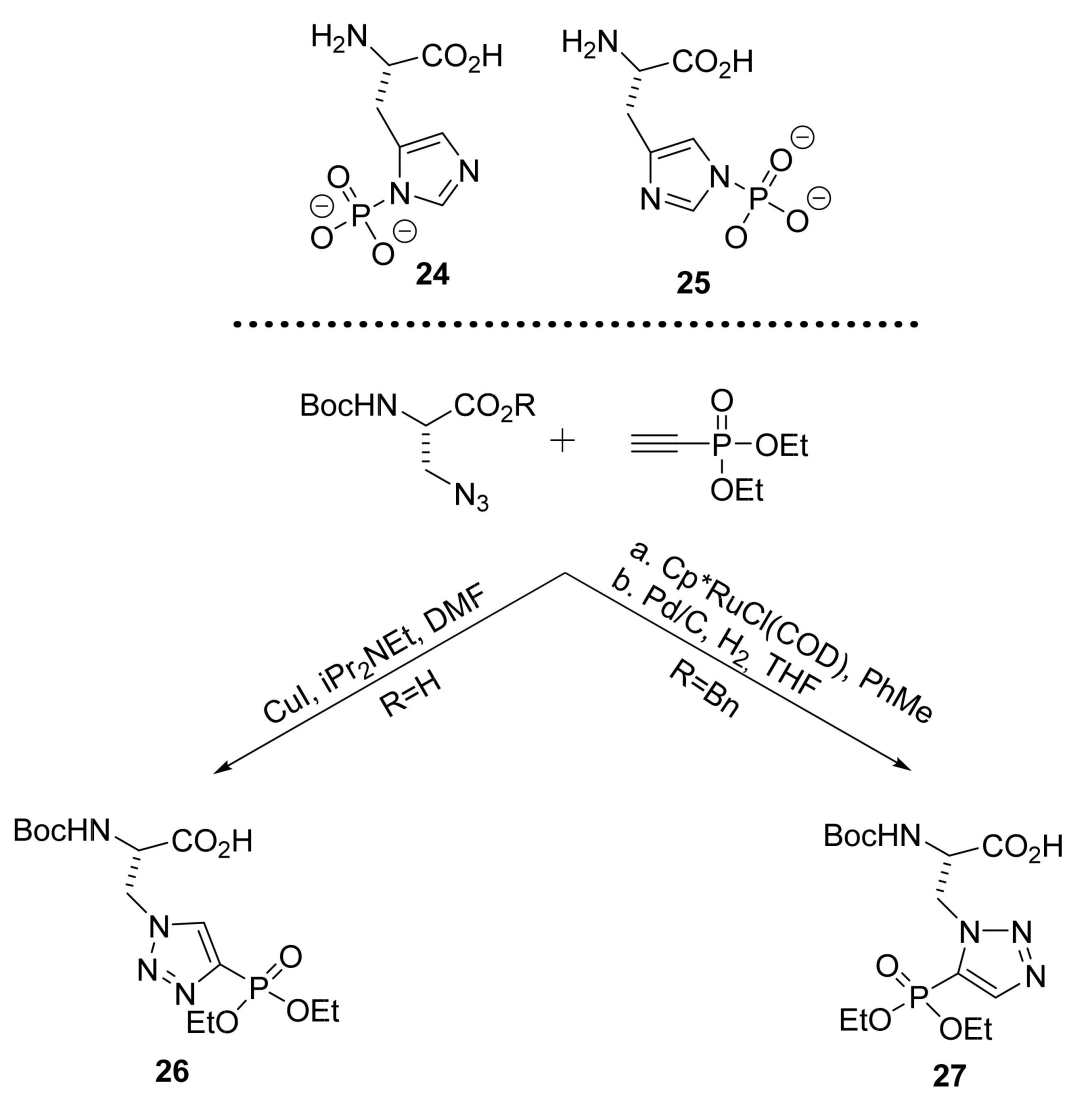
Synthesis of phosphorylated histidine analogues by CuAAC and RuAAC.

Protein kinases and protein phosphatases are important targets of drugs, because post-translational protein phosphorylation plays a key role in defining protein function. While the processes involved in the *O*-phosphorylation of serine, tyrosine, and threonine are well understood, the study of histidine phosphorylation is limited by the instability of histidine phosphorylated (His-p) and by the fact that there are two isomeric forms: **24** and **25** (Scheme 6).

In an attempt to overcome these problems, Kee et al. recently reported an easy method for synthesizing His-p **24** and **25** analogues using copper-catalyzed and ruthenium-catalyzed cycloadditions, respectively (Scheme 6) [52]. Mimetic **26** was incorporated into two different semi-synthetic proteins containing His-p to produce antibodies in the synthesized peptidomimetics. After the isolation of the produced antibodies, they selectively recognized the phosphorylated protein but not the non-phosphorylated counterpart.

The use of triazolic amino acids as small-molecule ligands for proteins is a relatively unexplored area of research [53,54]. Gajewski et al. investigated the use of these structures as a basis for neutral amino acid transport protein inhibitors (SN1) [53]. A superposition of the energy minimized the structures of the natural substrates (l-glutamine, l-aspargine, and l-histidine) of SN1, suggesting that an amino acid with a 1,4-disubstituted triazole in the side chain would be able to provide hydrogen bond interactions with the protein.

Fully protected triazole amino acids were prepared using the CuAAC of the β-azido-alanine with a range of acetylenes under standard conditions. Global deprotection gives amino acids that have been shown to be inactive at the SN1 transporter protein.

Stanley et al. [54] have used a similar approach to explore the potential of triazole amino acids as selective AMPA receptor ligands. Under standard conditions, 1,4-disubstituted triazolic amino acids and 1,5-substituted analogues were prepared using the CuAAC and the RuAAC methodologies, respectively.

**Figure 11:**
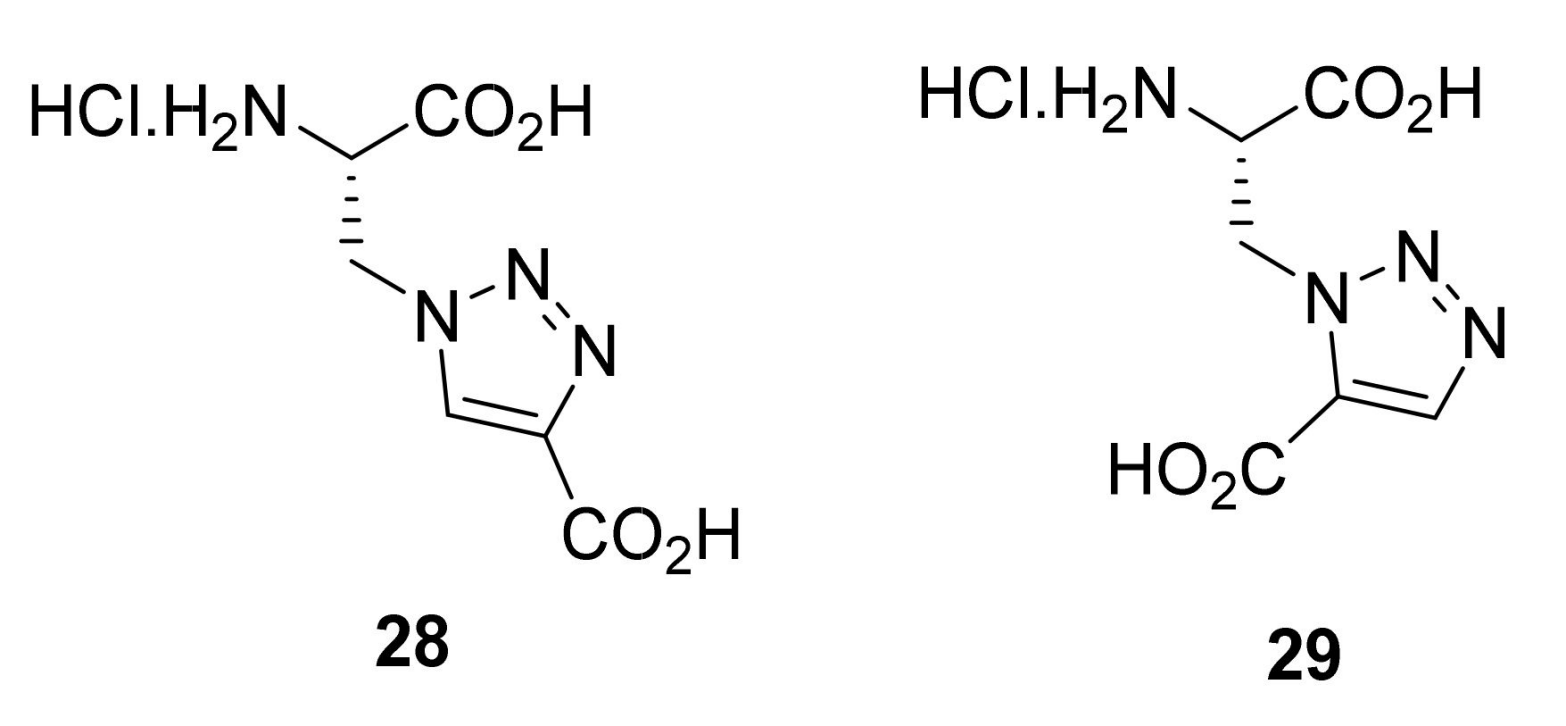
Two selective AMPA receptor ligands exhibiting competitive binding.

The synthesized triazole amino acids were tested in vitro on native ionotropic glutamate receptors (NMDA, AMPA, and kainate) and in functional assays of recombinant metabotropic glutamate receptors. Data from the in vitro competitive binding assays of ionotropic glutamate receptors revealed that **28** and **29** (Figure 11) were significant, although not greatly, competitive receptors, with IC_50_ values of 63 µM and 49 µM, respectively.

**Figure 12:**
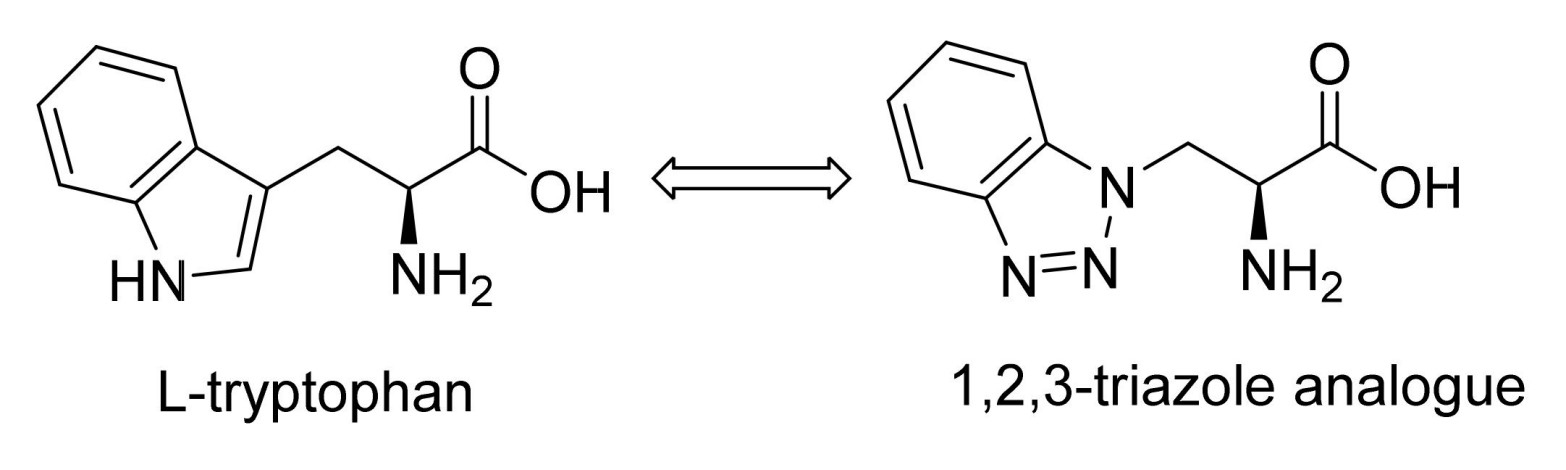
l-tryptophane and its triazole analogue.

A 1,2,3-triazole analogue of l-tryptophan [55] was recently synthesized using click chemistry conditions and tested for activity of the amino acid transporter type, LAT1. This analogue showed a better absorption rate than its corresponding tryptophan equivalent (Figure 12).

### 2.2. Cyclic Compounds

#### 2.2.1. “Head-to-Tail” Cyclization

Cyclic head-to-tail peptides occur in a wide variety of natural products, with an impressive range of biological activities [56,57]. These cyclic peptides generally improve metabolic stability and may improve structural rigidity due to the geometric preorganization into a suitable conformation for binding. As such, these structures are attractive for the design of small protein mimetics with a secondary structure. The head-to-tail cyclization, which is effective in forming cyclic peptides, is, however, quite difficult to achieve using conventional techniques.

**Figure 13:**
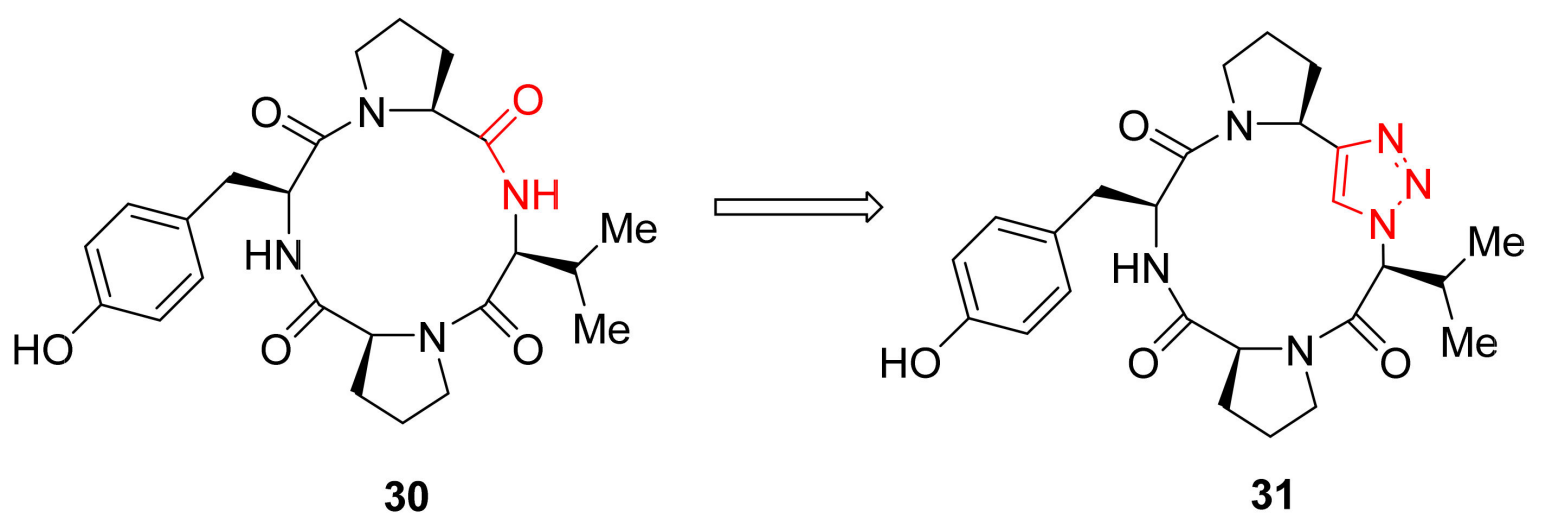
Substitution of the amide bond in the natural product **30** with 1,4-triazole.

The first report on cyclic triazolo-peptidomimetics was described by Ghadiri in 2003 [58]: 1,2,3-triazole 1,4-disubstituted was used as an amide bond isostere to form cyclic peptidomimetics that self-assemble into nanotubes. Bock et al. have published the synthesis of three highly tense cyclic tetrapeptide analogues (e.g., **31**, Figure 13) of tetrapeptide **30** (*cyclo*-[-l-Pro-l-Val-l-Pro-l-Tyr] of natural origin) using CuAAC [59].

**Figure 14:**
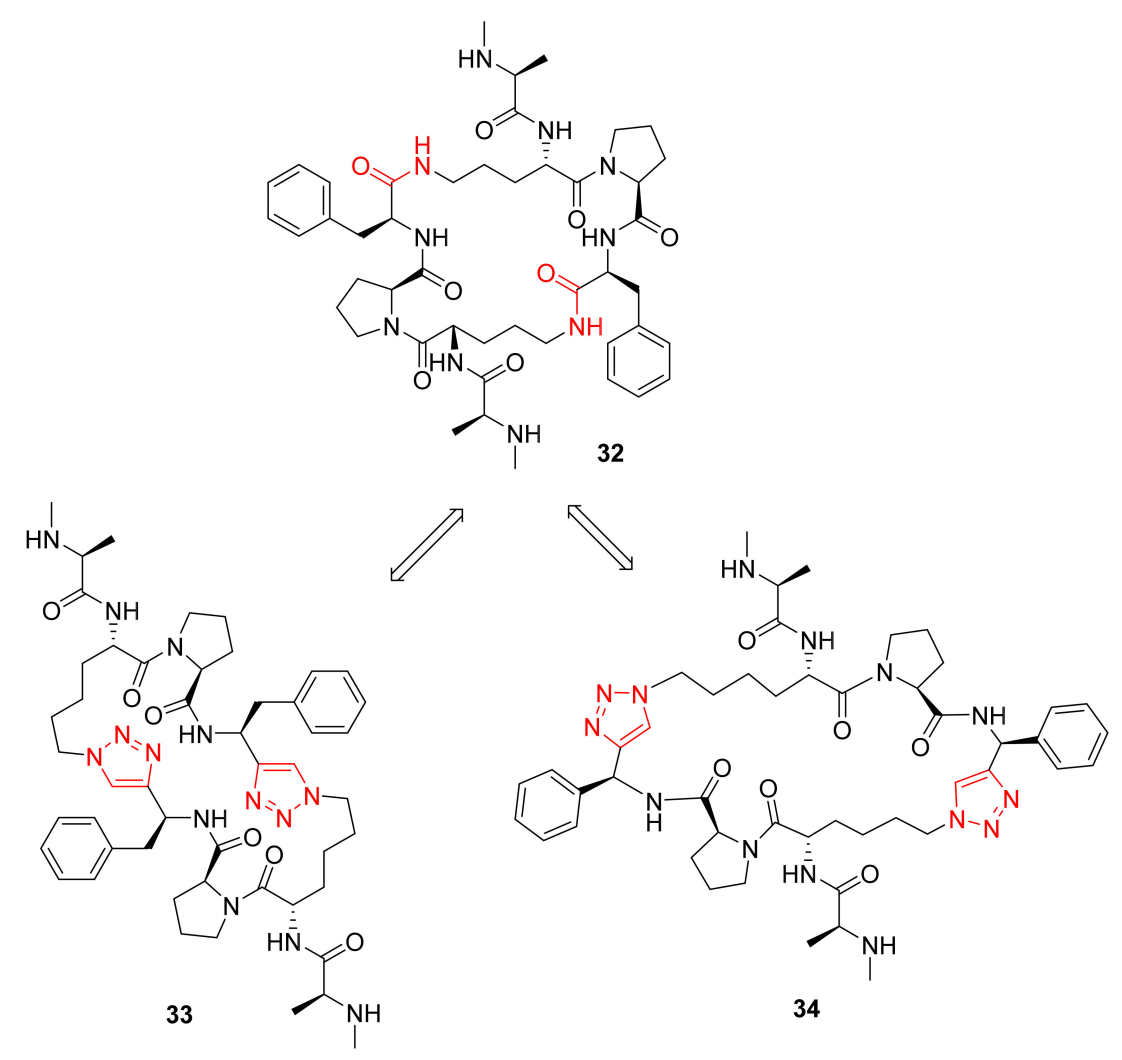
Structure of the Smac dimer **32** and its two derivatives, **33** and **34**.

Mitochondrial proteins, Smac and Smac mimetics, such as **32** (Figure 14), inhibit apoptosis and are therefore important anticancer agents. Wang et al. replaced the two amide bonds of **32** with two triazoles in an attempt to improve cell activity and reduce peptide characteristics [60]. 

CuAAC in liquid phase gave a linear mono-triazole dimer, which, after a new functionalization, was cyclized using CuAAC to produce the mimetic compounds, Smac **33** and **34**, after deprotection and derivatization. The new peptidomimetic agent, Smac **34**, is 5–8-fold more potent than the parent compound **32** in inhibiting the growth of cancer cells in two different cell lines: MDA-MB-231 (breast cancer) and SK-OV-3 cancer cell lines (ovarian cancer). 

**Figure 15:**
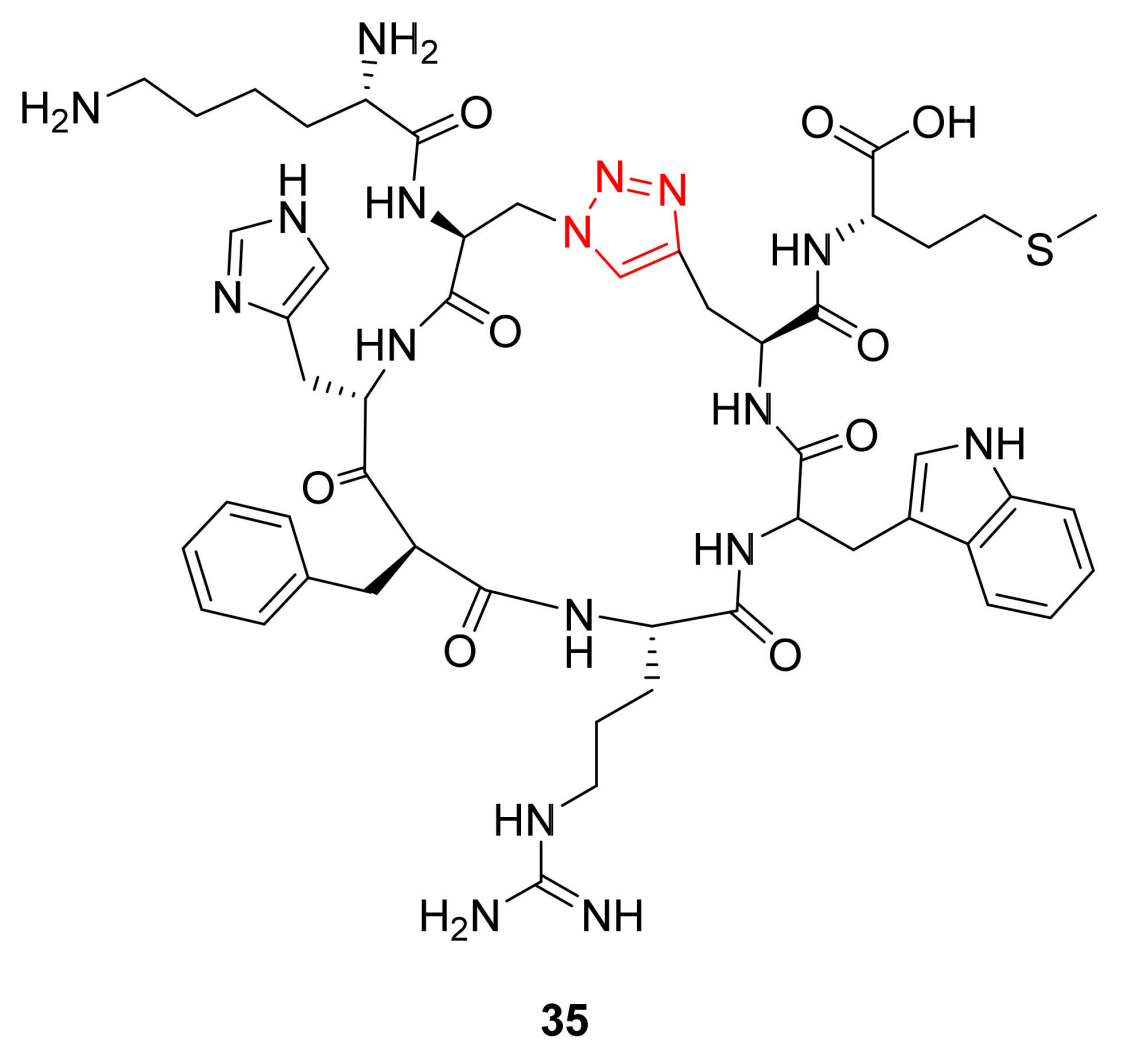
Cyclic peptide mimetic compound **35** containing a triazole.

Meldal et al. synthesized head-to-tail cyclic peptides **35** (Figure 15) by incorporating amino acids containing azide and alkyne into the standard synthesis of solid phase peptides based on Fmoc strategy [61]. Then, on-resin cyclization was applied.

Ghadiri et al. also described 1,4-disubstituted triazole-based peptidomimetics as ligands for the somatostatin receptor, SSTR [62]. Triazole was used to simulate the *trans*-peptide bond in a tetrapeptide. Sixteen chiral peptidomimetics were synthesized using CuAAC. Although the compound showed a high binding affinity (IC_50_ < 200 nM), its affinity was lower than the parent peptide, SRIF-28 (IC_50_ < 5 nM).

**Figure 16:**
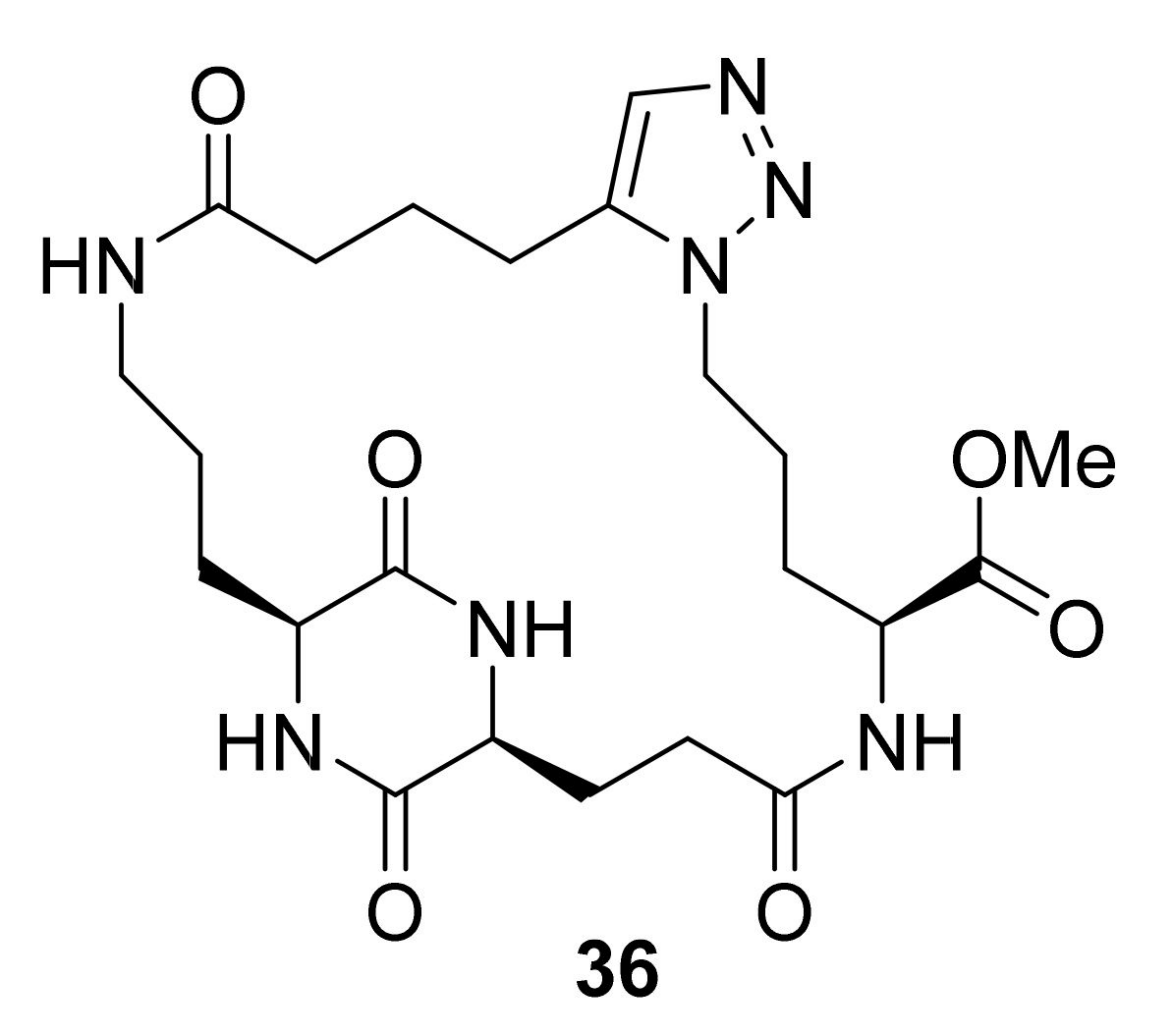
Example of peptidomimetic synthesis using the diversity-oriented synthesis (DOS) strategy.

Inspired by the large number of biologically active molecules containing peptide motifs, Spring et al. described the strategy of diversity-oriented synthesis (DOS) for the generation of macrocyclic peptidomimetic compounds [63]. DOS [64] is a synthetic strategy that allows for the production of molecule libraries with high levels of structural diversity and three-dimensionality. The resulting linear peptides, containing both a terminal alkyne and an azide, were used to prepare triazole-based macrocyclic peptidomimetics (an example of compound **36** is shown in Figure 16).

The same strategy (DOS) was used to synthesize indole/pyrimidinone triazole-based macrocyclic peptidomimetics [65]. The synthesis of the library starts with the preparation of alkyne-functionalized indoles and dihydropyrimidinones. Then, a four-component Mannich-type reaction is achieved to obtain the bromo-substituted amide derivative, alkyne. After the substitution of bromine with the azide moiety, an intramolecular copper (I)-catalyzed [3+2] azide–alkyne cycloaddition allows macrocyclic peptidomimetics with inhibitory property against CDK 2 to be obtained, which is a protein responsible for a variety of cancers.

**Figure 17:**
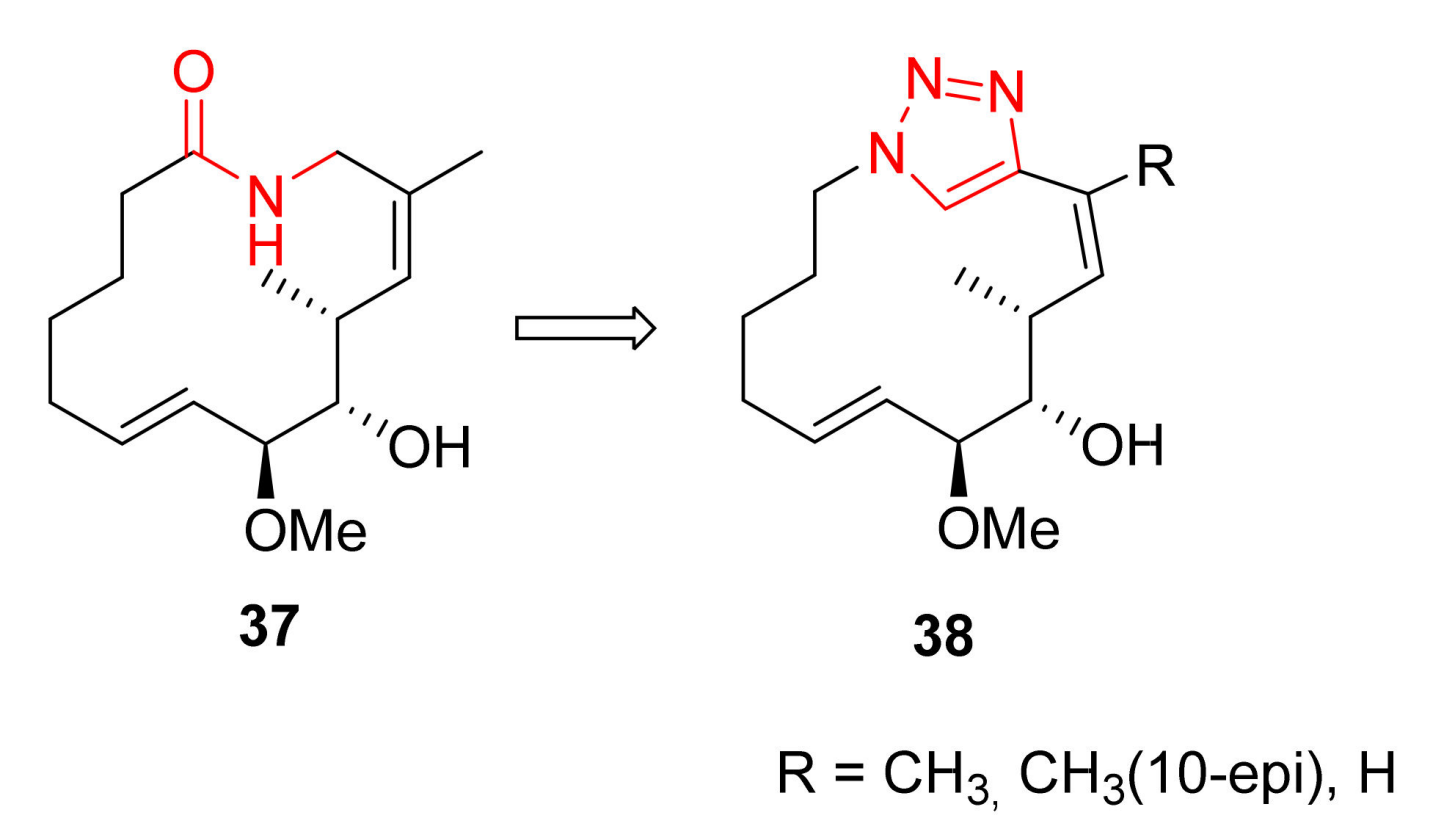
Structures of the migrastatin analogue **37** and triazole-migrastatine **38**.

The triazole moiety was also incorporated in macrocycle derivatives. Migrastatin is a natural product isolated from *Streptomyces platensis* bacteria and is an inhibitor of fascin, a protein involved in cell motility. The triazole analogues of an active lactam derivative of migrastatin **38** (Figure 17), synthesized using CuAAC, showed a significant effect in reducing the migration capacity of MDA-MB-361 cell lines [66]. 

Peptidomimetic derivatives based on sansalvamide A (San A) [67], an inhibitor of heat shock protein 90, are prepared by incorporating triazoles, oxazoles, thiazoles, or pseudoprolines along the macrocyclic backbone as amide bond surrogates. All the peptidomimetics were evaluated for their cytotoxicity, and the results showed that only triazole and thiazole can be incorporated without reducing the cytotoxicity. 

#### 2.2.2. Cyclization Side-Chain–Side-Chain

As mentioned above, a peptidomimetic with a triazole moiety has shown the structural stability of a coiled coil peptide. In addition, cyclization side-chain to side-chain by the copper-catalyzed dipolar cycloaddition in a VHP (villin headpiece) peptide shows this very well [68]. A VHP peptide has 36 residues that are stable in aqueous solution, with a compact tertiary folded structure composed of three α-helices. A side-chain to side-chain cyclization of this peptide stabilizes α-helices, and a cyclization via a triazole is the most stabilizing, compared to lactam and oxime. 

**Figure 18:**
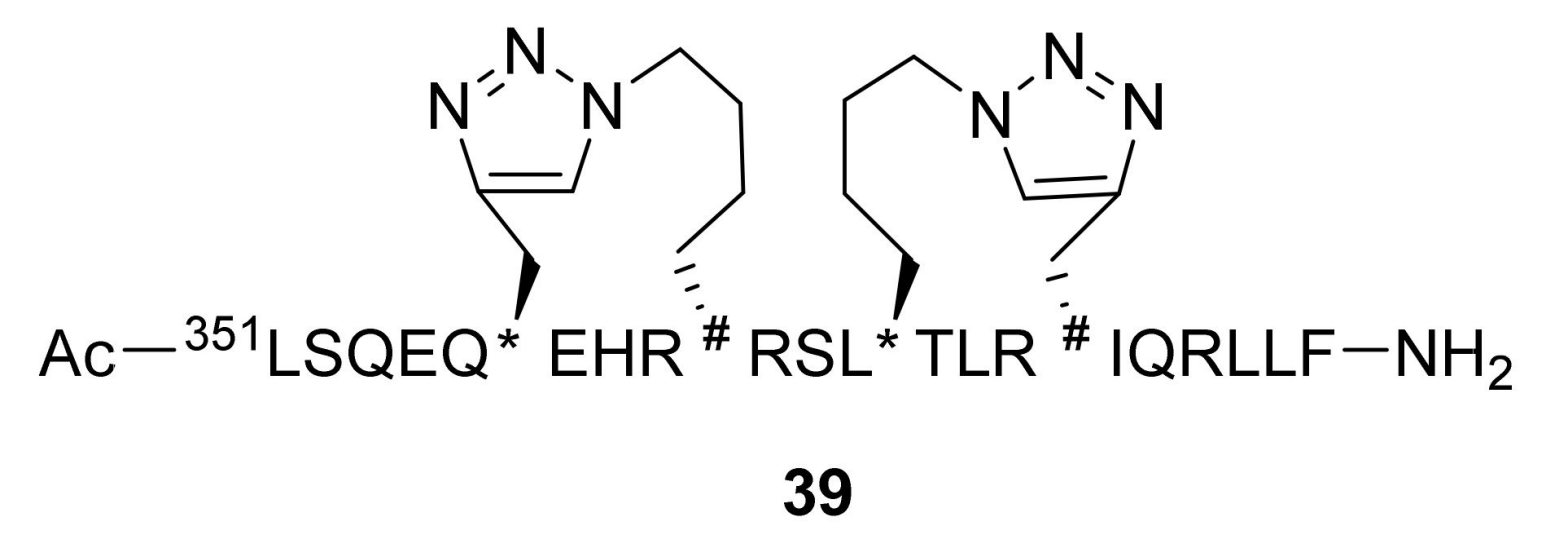
Stapled peptide targeting the β-catenin/BCL9 interaction. * indicates l-amino acid, and # indicates d-amino acid.

Kawamoto et al. also reported the use of CuAAC to generate peptides of α-helical BCL9 structures stapled with single or double triazole [69]. The triazole stapling method was used to stabilize the α-helical BCL9 peptides to target the BCL9/β-catenin protein–protein interaction. These peptides increased the rigidity and improved the helical structure. An example of double-stapled peptides (**39**, Figure 18) shows a 90% helical structure and a potent binding to β-catenin. Several of the designed single- and double-triazole-stapled peptides also showed a better resistance to proteolytic degradation.

Another example that also shows the stabilization of the structure is the i-to-i+5 side-chain to side-chain cyclization in Melanotan-II (MT-II) [70]. Cyclopeptides obtained by CuAAC cycloaddition of a type I β-turn structure gave potent melanocortin MT-II receptor agonists.

**Figure 19:**
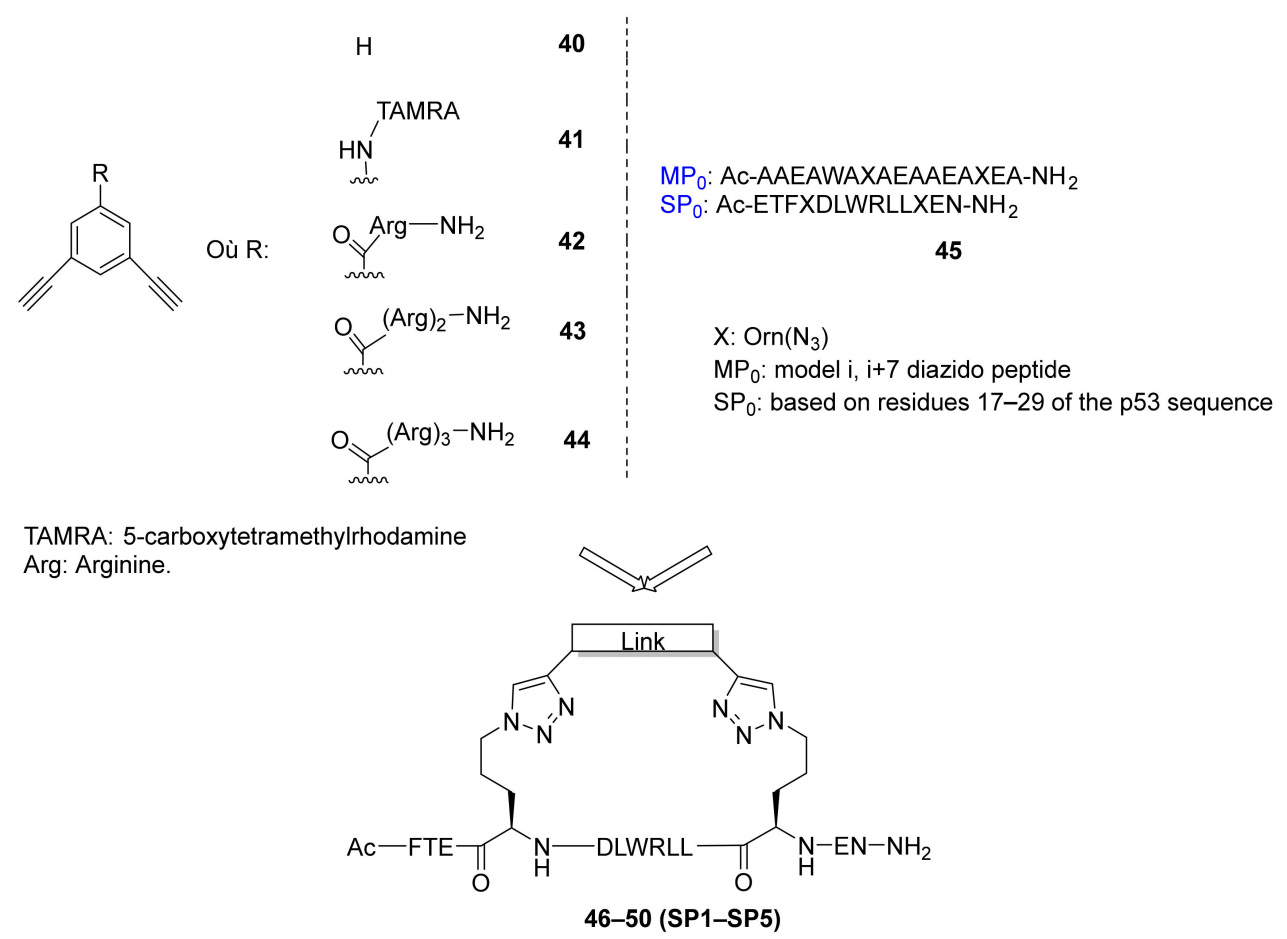
General structure of the stapled bis-triazolyl peptide product, SP1–SP5 (**46**–**50**).

Another side-chain to side-chain cyclization is the synthesis of “functionalized” stapled peptides **46**–**50**. Spring et al. [71] reported the synthesis of different functionalized peptides on the staple linkage from a peptide with two reactivity sites **45** (Figure 19) via a two-component strategy.

The triazole ring has shown its ability as an isostere of trans amide in the enzymatic macrocyclization. Generally, macrocyclization enzymes are active on peptide substrates. The PatGmac enzyme was able to macrocyclize peptide substrates containing one, two, and three 1,4-substituted 1,2,3-triazoles, and this shows that the triazole unit acts well as a *trans* amide isostere [72].

Triazole moieties prepared via CuAAC are interesting peptide isosteres. The copper(I)-catalyzed azide–alkyne cycloaddition reaction CuAAC has become a versatile and robust synthesis technique for the incorporation of 1,2,3-triazoles into peptide structures. This reaction has been used to achieve the following objectives:

- The synthesis of peptidotriazoles by the replacement of one or more amide bonds with triazole units;
- The preparation of β-turn secondary structures. Triazole-based compounds can mimic β-turn peptides, and triazole peptides can therefore be used for protein folding studies;
- The synthesis of triazolamer structures by replacing the amide bonds with 1,4-disubstituted 1,2,3-triazole;
- The replacement of a dipeptide within a multispiral α-helical secondary coiled coil structure;
- The modification of the amino acid side chain by the triazole motif to improve the stability or binding affinity through favorable interactions at the surface of the biological target;
- The macrocyclization of peptidomimetics in order to regulate the biological activity and stability of peptide macrocycles.

## 3. Compounds with Triazole Links to Other Features 

### 3.1. Peptide–Carbohydrate

Glycopeptides are natural or semi-synthetic products with a similar chemical structure that has a central nucleus of amino acids. Sugars are grafted onto this peptide nucleus. Now that glycopeptides are involved in various biological functions, they have side effects. Therefore, glycopeptides are not suicide inhibitors, and they form hydrogen bonds with the d-Ala-d-Ala motif of the peptidoglycan monomer, thus preventing the glycosidic chain elongation step. 

To overcome these problems, complex assemblies mimicking natural glycopeptides have been assayed, but they have been difficult to synthesize due to the difficulty of chemically analyzing carbohydrates and the sensitivity to hydrolysis of the glycosidic bonds. As a result, new methods for conjugating oligosaccharides into peptide sequences with more metabolically stable bonds have been developed. 

Van Der Wal et al. [73] synthesized a glycopeptide-based antifreeze polymer from glycopeptide azide and alkyne by the partial reduction of azide and polymerization by CuAAC to obtain linear oligomers.

In order to compare the activity with native antifreeze glycoproteins (AFGPs), a linear dodecapeptide **52** (oligomer with four repetitive units), comparable in length to AFGP-8 **51** (the AFGP glycoprotein with the lowest molecular weight found in nature), was synthesized.

**Figure 20:**
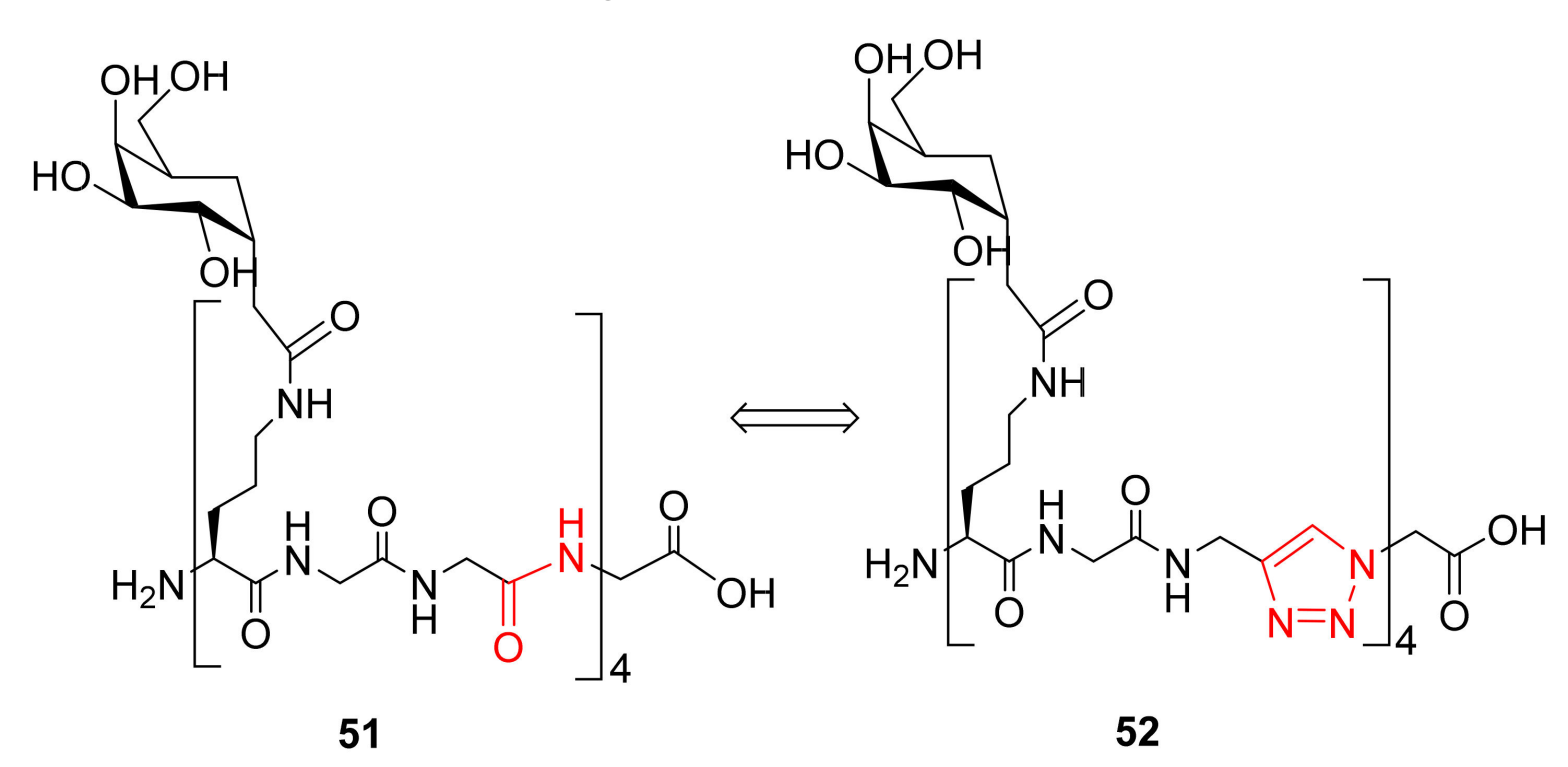
Comparison of AFGP **51** and its triazole-based tetramer mimetic **52**.

In terms of the ice recrystallization inhibition (IRI) activity, triazole oligomers showed a modest IRI activity, compared to AFGP-8; however, the CD spectroscopy showed that the triazole-based tetramer had a secondary structure which is similar to the amide-based tetramer based on AFGP-8 (Figure 20). 

Glycotriazole peptides derived from Hylasptin P_1_ (HSP1) [74], a 14-residue polypeptide, were prepared by solid phase peptide synthesis and CuAAC using peracetylated azide derivatives of glucose and *N*-acetyl-glucosamine and the protected side chain, [Pra^1^]HSP1-NH_2_-peptidyl-resin. In terms of comparing and studying the action of the carbohydrate ring, a non-glycosylated triazole analogue has been synthesized. The triazole derivatives showed a higher antifungal activity, compared to HSP1-NH_2_, and the carbohydrate motif further improved this antifungal activity. 

An efficient process for directly synthesizing glycomimetics has been developed [75]. It is a methodology allowing for the one-pot ligation of triazole glycopeptides. The stereoselective reaction uses 2-azido-1,3-dimethylimidazolinium hexaflurophosphate and CuAAC in one step. Two synthetic MUC1 alkyne peptides were glycoconjugated with GalNAc. This reaction is considered to be more efficient than a reaction in which glycosylazides are presynthesized. 

The Brimble group [76] synthesized mono- and di-glycosylated analogues of Pramlintide, which is an amylin analogue used in patients with types I and II diabetes. The synthesis of glycopeptide mimetics allowed the GlcNAc moiety to be incorporated at position 21, 35, or both by CuAAC. Using click chemistry, this glycosylation allowed for retention of the bioactivity of the receptor and improvement of the physico-chemical and pharmacokinetic properties. 

A new class of amphiphilic-conjugated aminoglycopeptides, called aminoglycoside-peptide triazole conjugates (APTCs), was prepared by the Schweizer group [77]. These APTCs are prepared using the CuAAC between a hydrophobic and ultrashort peptide alkyne, introduced as propargylglycine, and an azide derived from neomycin-B or kanamycin-A. Some APTCs have increased antibacterial activity against neomycin B- and kanamycin A-resistant strains. 

**Figure 21:**
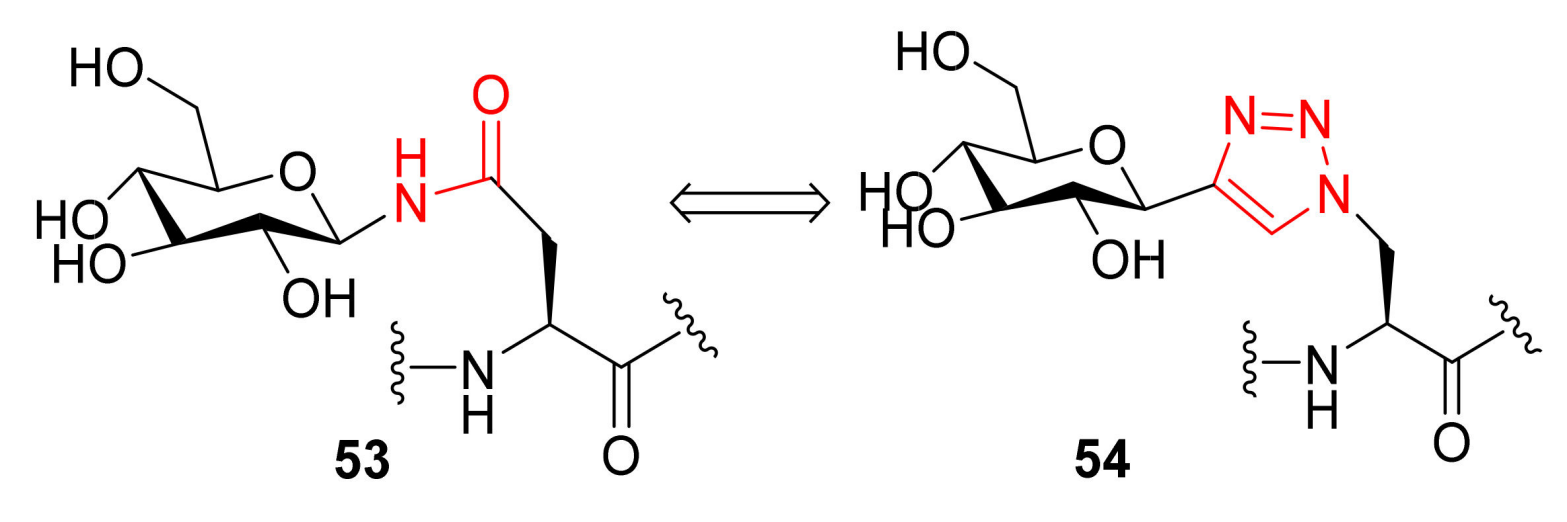
Triazole glycopeptide mimetics.

The amide bond in a glycoRGD peptide (arginine–glycine–aspartate) (**53**) that selectively binds to tumor-associated α_v_β_3_-integrin receptors was replaced by a mimetic triazole (**54**) without losing biological activity (Figure 21), and this shows the value of triazole as an amide isostere [78].

### 3.2. Peptide–Other Functions

Azide- or alkyne-modified peptides can be transformed into a variety of structures beyond glycopeptide analogues. Copper-catalyzed cycloadditions were used to add biotin and fluorescein.

**Scheme 7:**
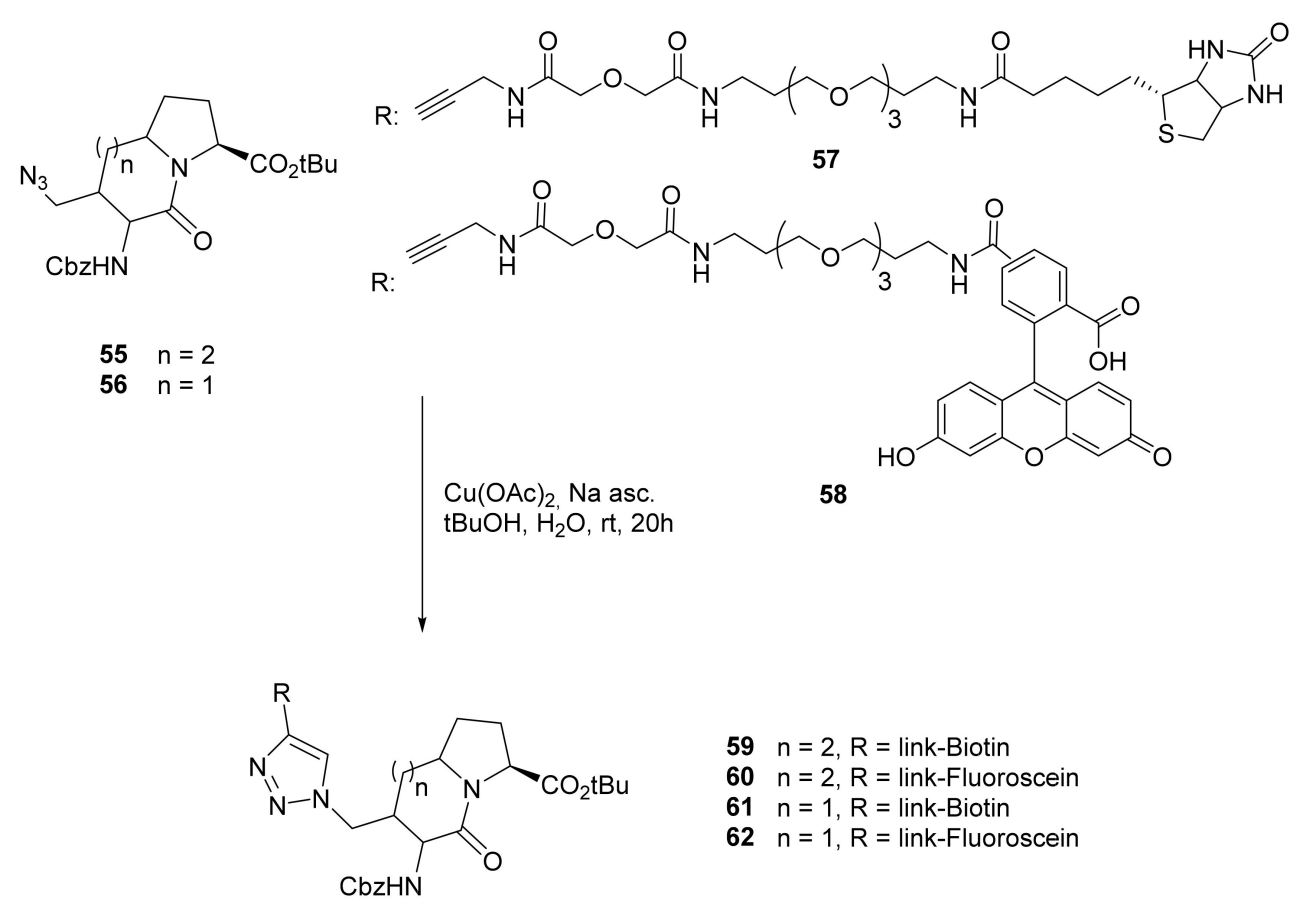
Cycloaddition between pseudopeptides **55** and **56** and conjugated biotin and fluorescein.

The 1,3-dipole cycloaddition reaction between pseudopeptides **55** and **56**, prepared by the introduction of the azide group into the azabicycloalkane amino acid, the conjugated biotin **57**, and the conjugated fluorescein **58** was performed, using Cu(OAc)_2_/sodium ascorbate as a catalyst in a 1:1 mixture of t-BuOH/H_2_O (Scheme 7) [79].

**Scheme 8:**
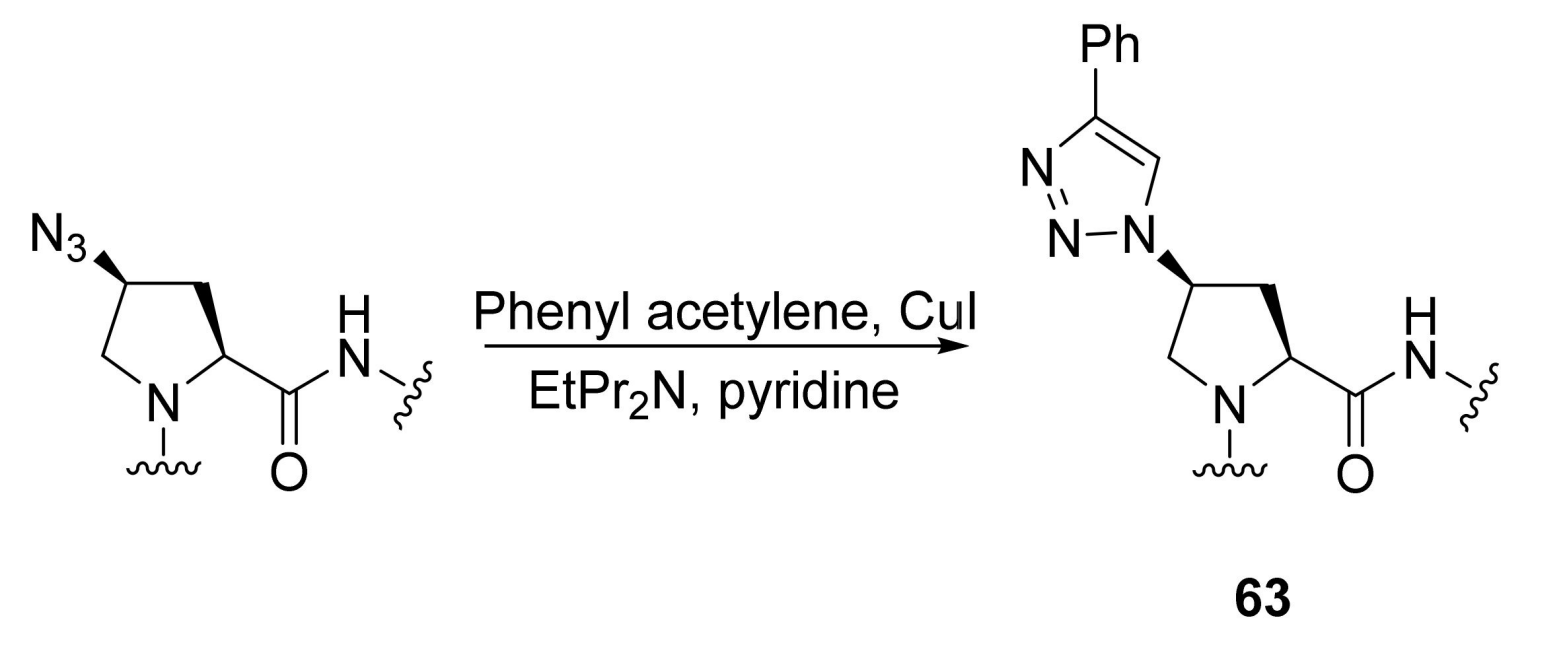
Synthesis of a peptide with substituted proline.

The residue pro 6 in dodecapeptide RINNIPWSEAMM was substituted by *cis*-4-azidoproline to allow for the preparation of a series of analogues via CuAAC reactions with azides [80]. Among the synthesized triazole peptides was the 4-phenyl-substituted triazole **63** (Scheme 8), which has a high affinity to envelope the glycoprotein, gp120, of HIV-1. This is explained by the fact that the phenyl is oriented in a more rigid position due to the triazole ring. Therefore, gp120 binding requires a lower entropic penalty, compared to the peptide, where the γ-aminoproline is coupled to the benzoyl group.

**Figure 22:**
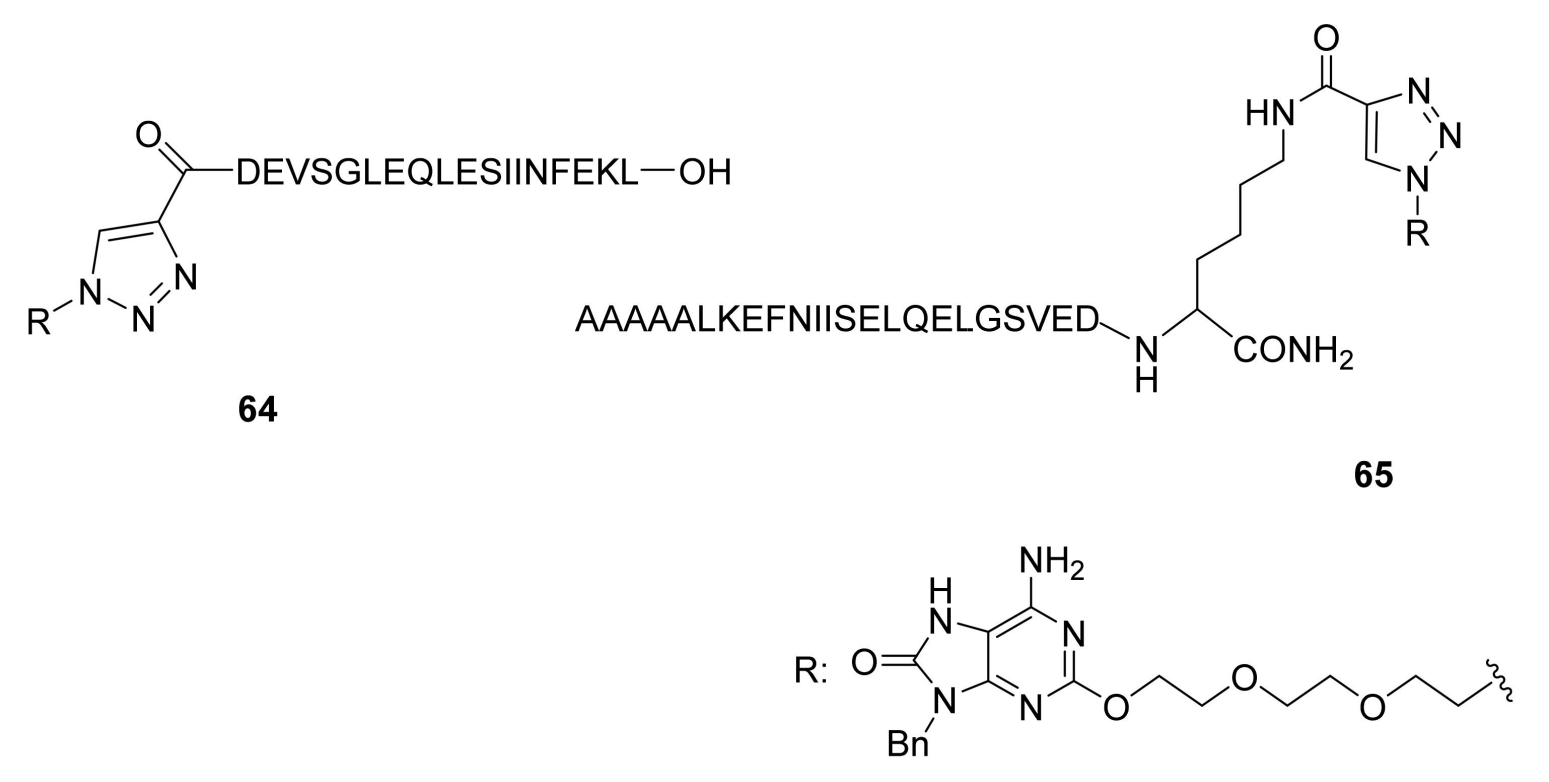
Triazole peptide derivatives with conjugated 2-alkoxy-8-hydroxyadenine.

One of the simpler and more general approaches is to link propiolic acid to the N-terminal or side-chain of the lysine or ornithine residues and then make changes through the CuAAC process. This was achieved, for example, on peptide chains containing the sequence, SIINFEKL (major histocompatibility complex MHC class I), to access derivatives **64** and **65** with conjugated 2-alcoxy-8-hydroxyadenin entities (Figure 22) [81].

### 3.3. Peptide–Polymer and Dendrimer

Peptide dendrimers have attracted a strong interest in science and technology and are widely applied in the field of biomedicine and technology [82,83].

**Figure 23:**
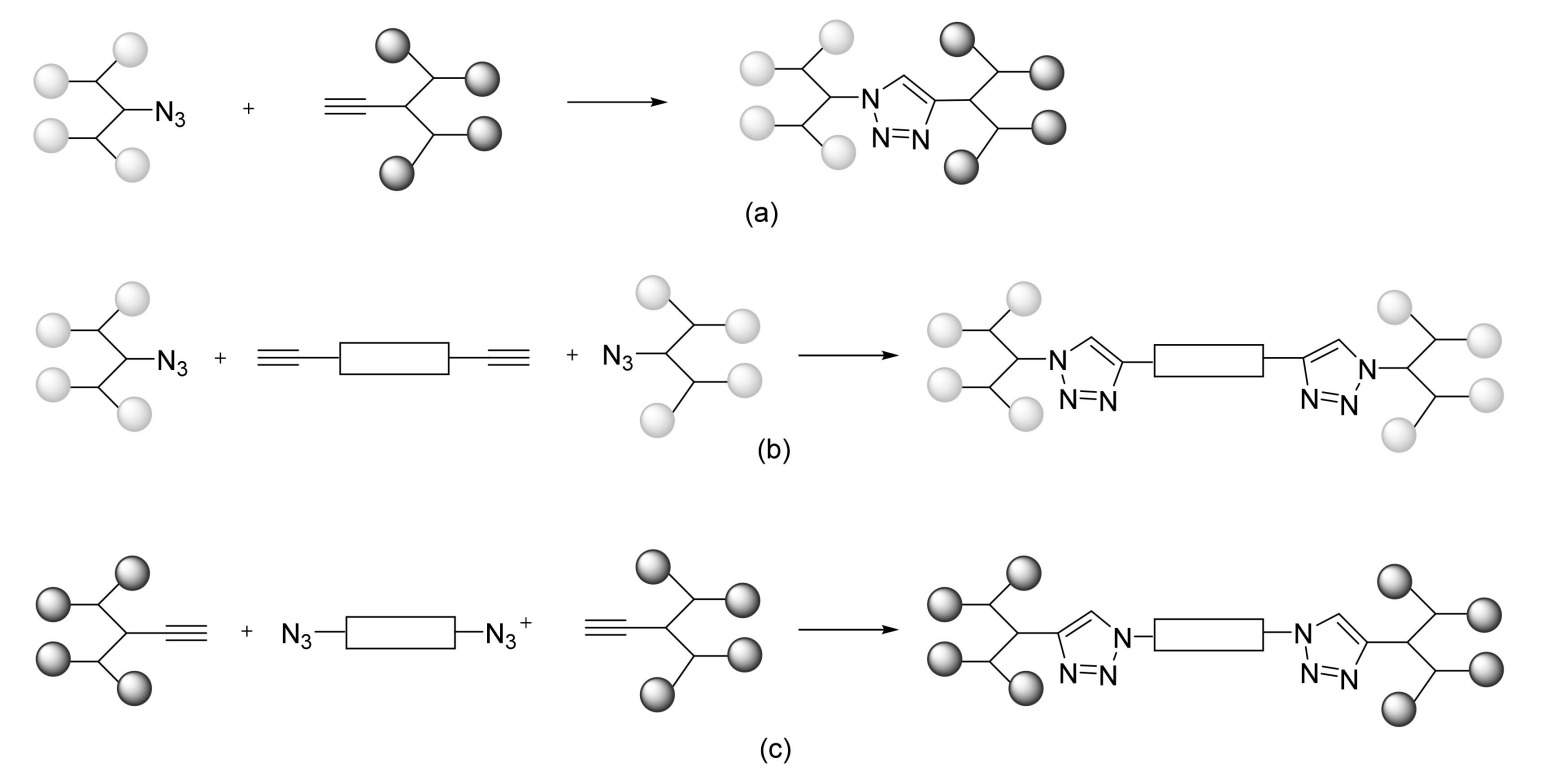
Strategy for the synthesis of peptide dendrimers using click chemistry. (**a**): CuAAC of an alkyne anchored in one dendron and an azide in another dendron; (**b**): CuAAC of a dialkyne nucleus and dendrons functionalized with azide units; (**c**): CuAAC of a diazide nucleus and dendrons functionalized with alkyne units.

Haridas et al. [84] synthesized a series of symmetric and asymmetric peptide dendrimers with a complex architecture using the CuAAC reaction. Three synthesis strategies were used (Figure 23a–c): the first (a) was to anchor the alkyne in one dendron and the azide in the other dendron. These dendrons were then linked using click chemistry reactions to obtain the dendrimers. In the second strategy (b), a dialkyne nucleus was chosen and the dendrons, functionalized with azide units, were coupled to give the dendrimer. The third strategy (c) was to pair dendrons, functionalized with alkyne units, and the azide nucleus.

The same Haridas group [85] synthesized urea–triazole-based peptide dendrimers. A urea-based dialkyne was reacted with azide-equipped dendrons by cycloaddition in the presence of Cu(I). The same dendrimers—based on urea but not triazole—were also synthesized. The two series of peptide dendrimers with urea and urea–triazole cores showed two different morphologies: fibrous for urea core dendrimers and vesicular for urea–triazole core dendrimers. This demonstrates the influence of the presence of triazole in the core unit on the self-assembly. 

Recently, the same group [86] prepared a series of peptide dendrimers based on aspartate and glutamate by CuAAC. Some synthesized products exhibit excellent gelation in DMSO, which shows a strong self-assembly.

A new synthesis methodology for two-block copolypeptides was described by Taton et al. [87], combining the ring-opening polymerization (ROP) of *N*-carboxyanhydrides (NCA) with CuAAC. Poly(γ-benzyl-l-Glutamate) (PBLGlu) and poly(trifluoroacetyl-l-Lysine) (PTFALys), each containing either an alkyne or azide functional group, were first synthesized by the ROP of the corresponding NCA at room temperature in DMF using bifunctional initiators containing α-alkyne and α-azide. Azide and alkyne polymers were then conjugated in DMF at 50 °C, with CuBr complexed by *N*,*N*,*N*’,*N*’’,*N*’’-pentamethyldiethylenetriamine (PNDETA) as a catalyst. After 36 h, block polymers were obtained with an almost-quantitative yield. 

Sureshan et al. [88,89,90,91] used another method of polymerization: topochemical azide–alkyne cycloaddition (TAAC) polymerization. This reaction makes it possible to synthesize triazolic peptidomimetics with repetitive sequences, without a catalyst and without a solvent. A dipeptide-monomer containing azides and alkynes at its termini was synthesized by coupling an azidoamino acid to an amino acid propargylamide and then crystallizing it from a mixture of toluene and hexane. The molecules of this monomer are in a parallel β-sheet arrangement in one direction, and a head-to-tail arrangement in a direction perpendicular to the β-sheet-direction. This anti-parallel arrangement places azide and alkyne in a reaction. The crystals of this monomer, upon heating to 85 °C, undergo crystal-to-crystal topochemical azide–alkyne cycloaddition (TAAC) polymerization to yield 1,4-triazole-linked oligopeptides. 

### 3.4. Conjugated Metal Complexes for Radiolabeling

**Figure 24:**
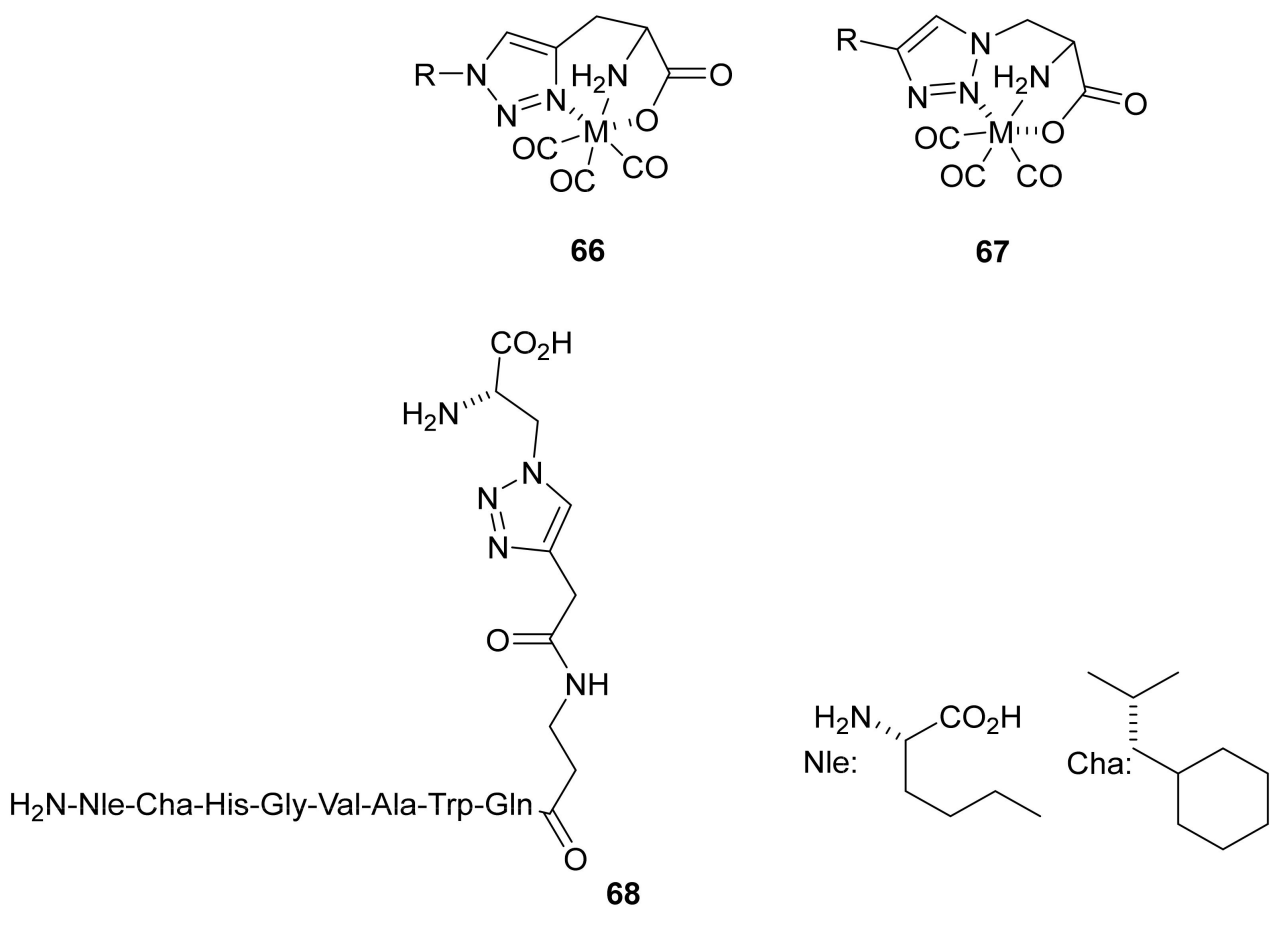
Metal complexes **66** and **67** obtained from the triazole peptide derivative **68**.

Triazoles are good ligands for transition metals. Peptides may be C- or N-terminated with an amino acid-derived azide or alkyne, which is used for CuAAC. This procedure addresses two objectives simultaneously. Firstly, it introduces a metal binding site on the peptide. Secondly, the triazole is itself part of the metal ligand. An obvious application of this approach is in the labeling of bioactive peptides for in vivo or cellular imaging. Type **66** and **67** complexes (Figure 24) have been prepared, where triazole units have different orientations but are always willing to coordinate with metals. The technetium complexes of the derived peptide **68** (Figure 24) were prepared for this purpose [92]. The synthesis and radiolabeling of biomolecules were conducted in a single step directly from [^99m^TcO4]^−^, which was added to the IsoLink^TM^ Kit. The triazole ligand was then added. This method of synthesis is highly desirable for potential clinical applications. 

An example of radiolabeling where triazole is part of the radiolabels is the preparation of ^99m^Tc(CO)_3_-labeled cRGDfK peptide analogues [93]. A CuAAC reaction was carried out between the cRGDfK peptide conjugated with N_3_-PEG_7_-COOH/N_3_-CH_2_-COOH and propargyl glycine in the presence of copper sulfate and sodium ascorbate, and subsequently, the peptide analogues were radiolabeled by ^99m^Tc using the [^99m^Tc(CO)_3_(H_2_O)_3_]^+^ precursor.

**Figure 25:**
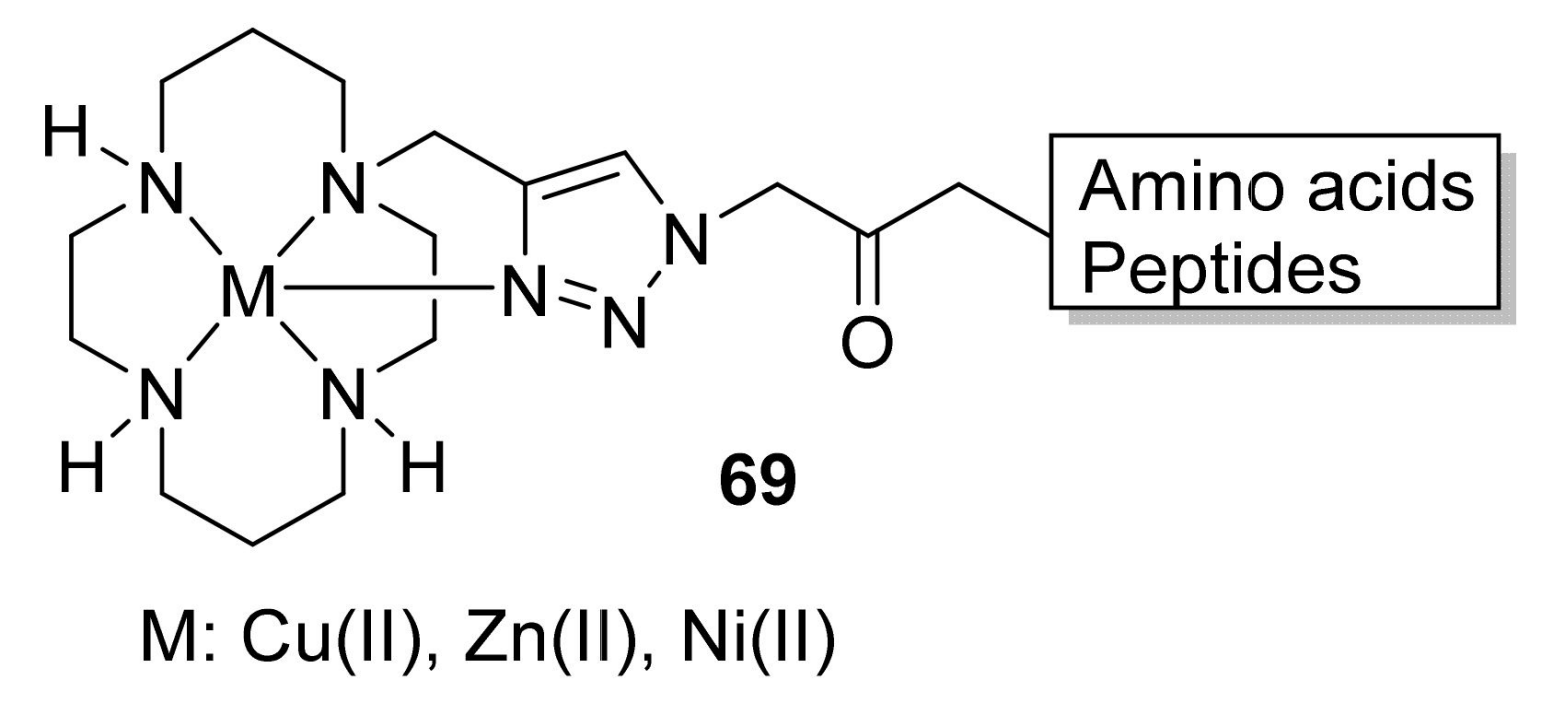
Cyclam metal complex derivatives.

Another example is the synthesis of derivatives of cyclam metal complex **69** with amino acids or a tripeptide using the CuAAC reaction (Figure 25) [94]. 

**Scheme 9:**
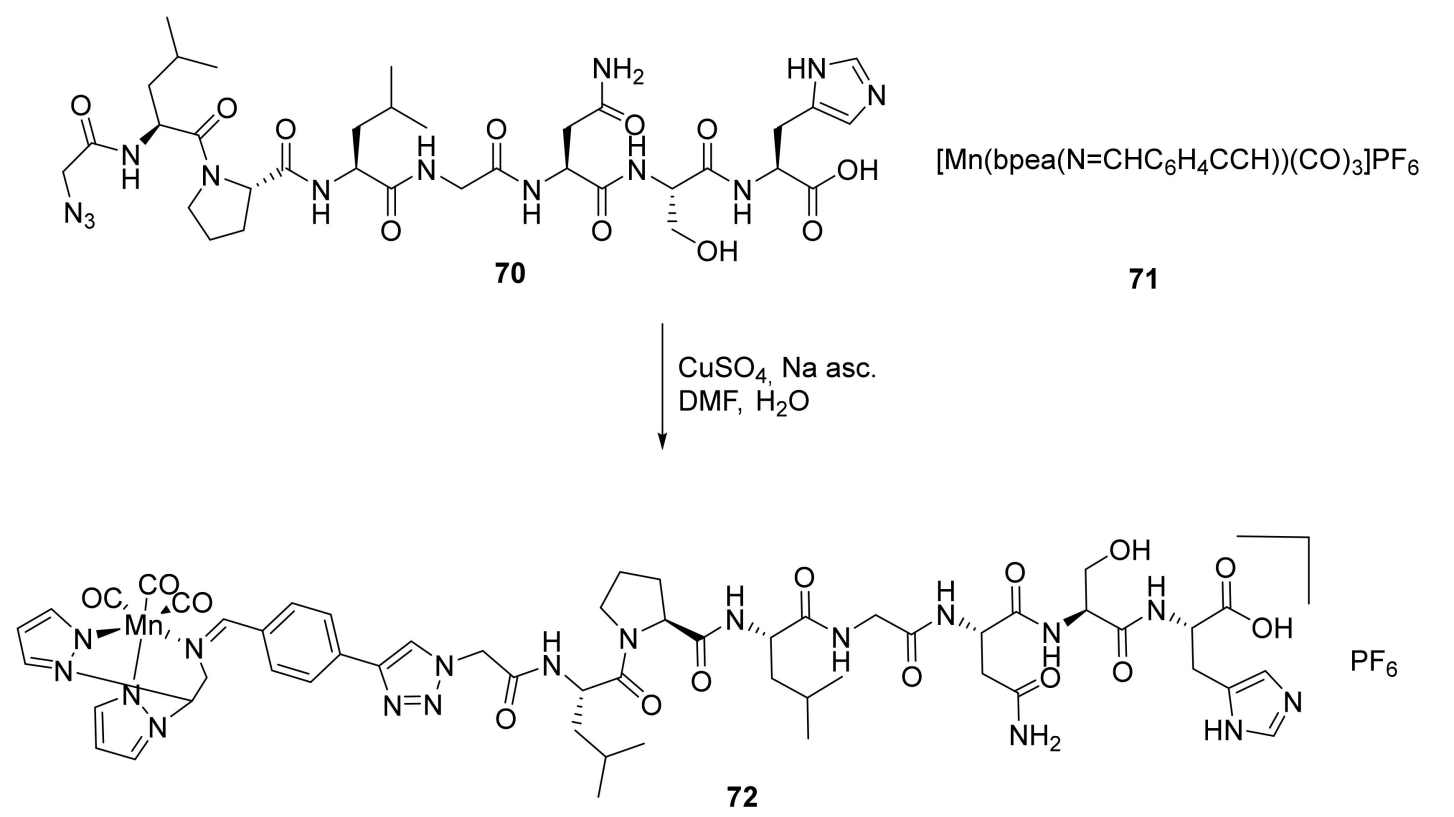
Synthesis of conjugated peptide **72** by CuAAC.

Schatzschneider et al. [95] synthesized a new conjugated triazole peptide from the tricarbonylmanganese(I) complex containing the 2,2-bis(pyrazolyl)ethylamine (bpea) ligand. Derivative **72** was prepared by CuAAC between the complex [Mn-(bpea(N=CHC_6_H_4_CCH))(CO)_3_]PF_6_ (**71**) and the peptide sequence N_3_-Ac-Leu-Pro-Leu-Gly-Asn-Ser-His-OH (**70**) in a mixture of DMF and water and in the presence of copper sulfate pentahydrate and sodium ascorbate (Scheme 9).

The Mindt and Behe group [96,97] prepared [NLe^15^]MG11 minigastrin analog triazolo-peptidomimetics labeled with ^177^Lu. They introduced 1,4-disubstituted 1,2,3-triazoles as bioisosteres to replace three amide bonds of peptidic radioconjugates with improved activity and tumor-targeting properties. 

Similarly, a peptidomimetic conjugate of ^177^Lu-labeled neurotensin was synthesized by substituting two amide bonds with triazoles into the backbone, thus preserving biological function [98].

This further endorses the advantage of the substitution of amides by triazoles in the development of peptidomimetics with improved properties.

CuAAC is an excellent method for linking peptides to other functional moieties. CuAAC is used:

- To conjugate carbohydrates into peptide sequences with more metabolically stable bonds;
- To link peptides to functions that will be subsequently oriented in more rigid positions;
- To add biotin and fluorescein;
- In the synthesis of functional peptide polymer and dendrimer with improved properties;
- In the preparation of conjugated metal complex peptidomimetics for radiolabeling, which is highly desirable for potential clinical applications using triazole as a bioisostere to replace the amide bond or as a tool to conjugate complex metal ions to peptides.

## 4. 1,2,3-Triazoles in Other Mimetics

### 4.1. Peptoids 

Peptoids are oligomers of N-substituted glycine units. The interesting feature of peptoids is that they are formed from primary amines, and a wide selection of them are commercially available. Peptoids are relatively easy to synthesize, more proteolytically stable than peptides, and have a significant potential for use in many different medical applications [99,100].

**Scheme 10:**
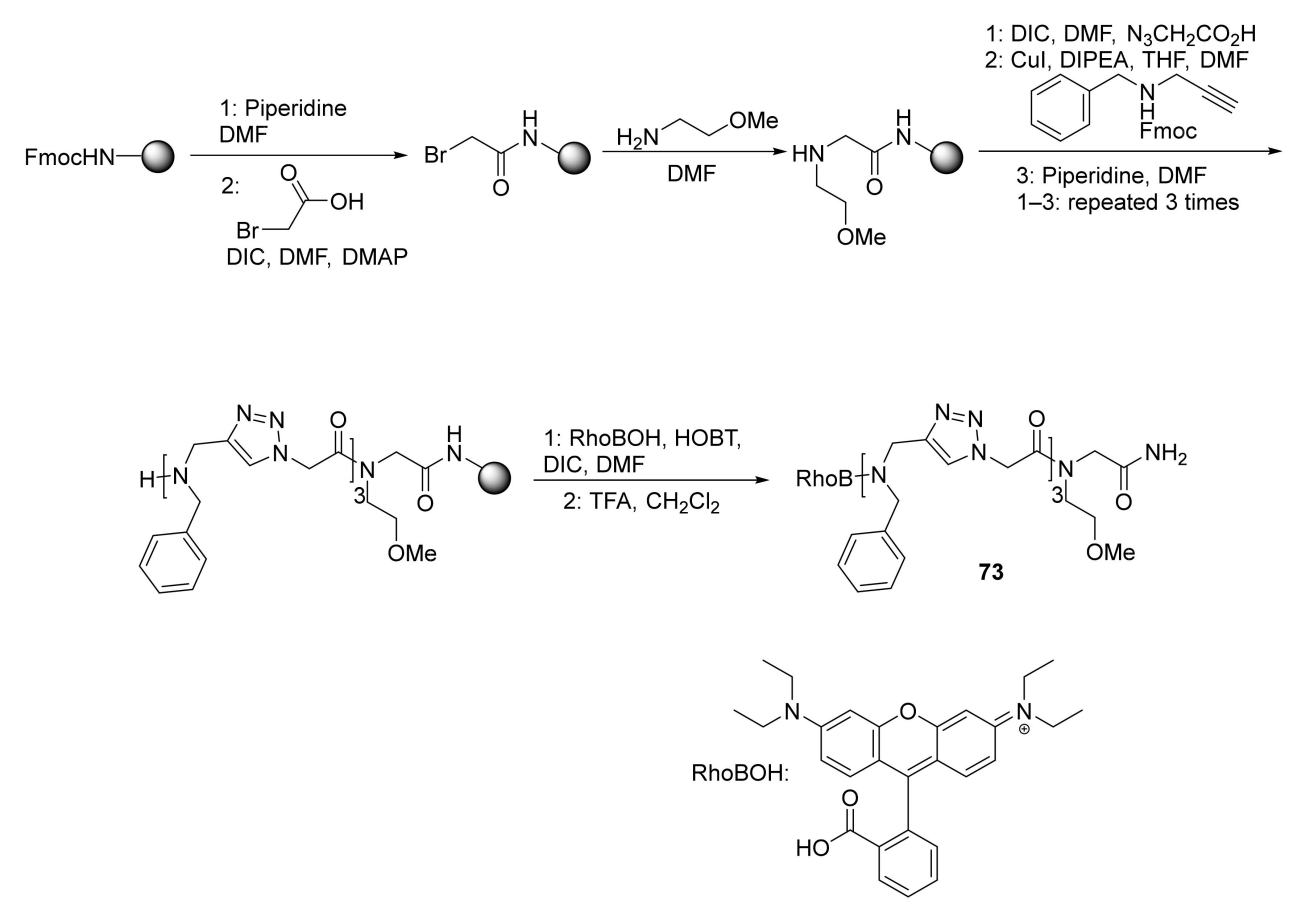
Example of synthesis of 1,4-triazolopeptoid in solid phase.

Althuon et al. [101] synthesized linear 1,4-triazolopeptoids (e.g., **73**) as a new class of cell-penetrating peptidomimetics. Repetitive triazole fragments with up to three residues were assembled on solid supports using CuAAC (Scheme 10). The 1,4-triazolopeptoides were labeled with rhodamine B as a fluorophore.

Biological tests on zebrafish embryos have shown that 1,4-triazolopeptoides can function as molecular transporters.

Cyclic peptoids have many advantages over linear peptides. They have an excellent cell permeability [102] and the ability to form more stable secondary structures [103].

Recently, a synthesis of a Meta596-Conjugate [104] confirmed the improvement in cyclic peptoid stability. Meta596 is a cyclic peptoid with three proline residues and three N-substituted glycine residues, two of which are N-propargyl peptoid residues. CuAAC occurred between the two side-chain alkyne groups and N-terminal azido groups of two GCNshSN peptide chains to provide the Meta596-Conjugate. The two Meta596-Conjugate-related peptides assembled into a folded coiled coil structure and exhibited a greater thermostability than the coiled coil formed by unconjugated GCNshSN peptides.

Additionally, combining peptide cyclization with peptoids improves the pharmacological properties of peptides [105,106,107,108]. Cyclic peptide–peptoid hybrids synthesized from HIV-1 integrase (IN)-derived peptide IN_181–188_ have an improved biological activity and high stability, compared to linear peptides. Click chemistry optimized by microwave-assisted automate solid phase synthesis (MW-SPPS), a rapid method lasting only a few hours, enabled a library of cyclic peptide–peptoid hybrids to be obtained [109].

Salvador et al. [110] prepared linear peptoids using an Ugi four-component reaction (U-4CR) and their macrocyclic analogues by CuAAC cycloaddition, generating the structure of the peptidomimetic nucleus. The methodology used included the continuous formation of an isocyanide, as well as of an azide-functionalized carboxylic acid, which are then used in U-4CR. The linear peptoids obtained could then be cyclized by CuAAC. The resulting convergent synthesis was characterized by an overall reaction time of 25 min, generating the desired peptidomimetics with good to excellent yields.

### 4.2. Non-Peptide Mimetics in Which Triazoles Replace Amides 

In recent years, the replacement of the amide bond with triazole has become a powerful tool for the synthesis of bioactive molecules, with improved biological, pharmaceutical, and physico-chemical properties.

The bioisosteric replacement between amide bonds and 1,4-disubstituted 1,2,3-triazole provides new analogues of benznidazole with anti-trypanosomal activity [111]. These compounds were synthesized by CuAAC and designed as a Craig-plot (two-dimensional plot) and Topliss Scheme (decision-making tree). The obtained 1,2,3-triazoles derivatives with aryl groups had similar or higher anti-trypanosomal activity than benznidazole. 

The 1,2,3-triazoles can replace all the amide bonds at the organogelator C_12_-Cyc (*N*,*N*’-((1S,2S)-cyclohexane-1,2-diyl)didodecanamide), to give the click-C_12_-Cyc while preserving the gelation properties [112]. In general, the antiparallel hydrogen bonds of the two C_12_-Cyc amides are self-assembled, and under the effect of van der Waals intermolecular interactions, gelation occurred. Therefore, gelation maintained in the case of substitution by triazoles was not obvious. This shows that triazoles take up all functions derived from amides. Click-C_12_-Cyc is characterized by non-cytotoxicity and a selective phase gelation of water–oil mixtures.

In 2019, a 1,2,3-triazole-conjugated phenacetin molecule (PhTC) was synthesized [113]. Phenacetin is an analgesic that was withdrawn from the market in 1983, because it is nephrotoxic and carcinogenic. The amide bond which is the cause of phenacetin toxicity was replaced with 1,2,3-triazole. Pharmacological assays showed the superior anti-inflammatory, anti-nociceptive, and anti-pyretic potential of the conjugate in comparison to the popular non-steroidal anti-inflammatory drugs (NSAIDs). In addition, the bioisosteric replacement of the amide bond in phenacetin by 1,2,3-triazole yields a conjugate with a superior efficacy and reduced toxicity. 

*N*-acetyl-paminophenol (AP) (acetaminophen), also called paracetamol, is the most widely used and prescribed analgesic and antipyretic in the world. Its most frequently reported adverse side effect is hepatotoxicity. To reduce this toxicity, 17 acetaminophen-triazole derivatives (APTDs) were synthesized by bioisosteric replacement of the amide bond of AP with 1,2,3-triazole [114]. CuAAC allows 4-(4-Methyl-1*H*-1,2,3-triazol-1-yl)phenol and tert-Butyl(4-((1-(4-Hydroxyphenyl)-1*H*-1,2,3-triazol-4-yl)methoxy)phenyl)carbamate to be obtained, which are two derivatives that have more powerful anti-inflammatory, analgesic, and antipyretic activities, without adverse events in either acute or sub-toxicological analyses.

**Figure 26:**
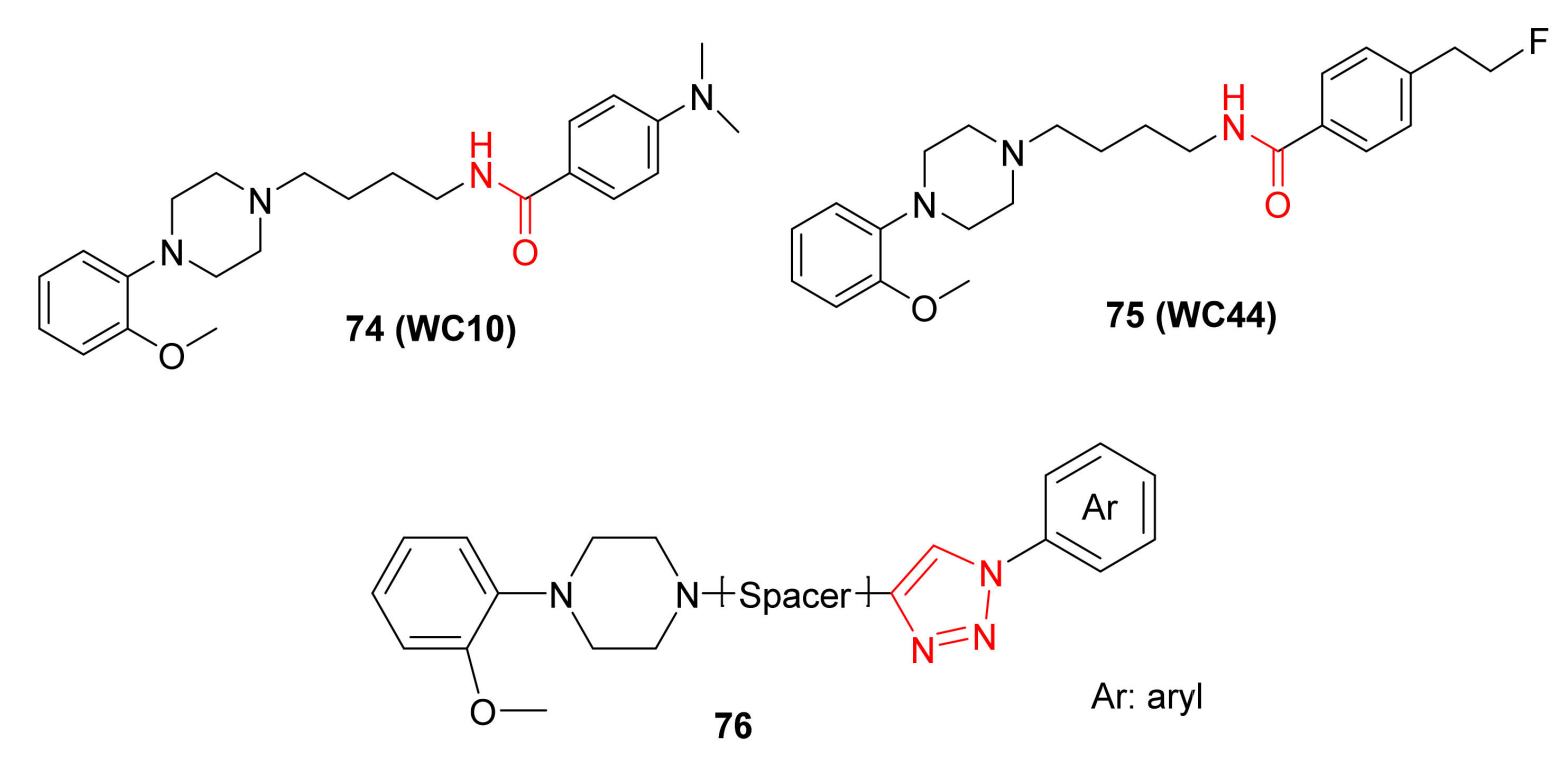
Structure of the compounds WC 10 and WC 44, and their triazole analogues.

Peng et al. [115] synthesized 2-methoxyphenyl piperazine analogues containing a triazole ring **76** (Figure 26) by replacing the amide bond in the WC series (**74** and **75**) [116]. Some prepared compounds showed a high affinity for dopamine D3 receptors, a neurotransmitter that plays several important roles in the central nervous system, and a moderate selectivity of dopamine D3 with respect to the D2 receptor subtypes. The target compounds were synthesized using CuAAC.

The Altimari group [117] prepared 1,4-substituted 1,2,3-triazole mimetics derived from bicalutamide for new prostate cancer treatments. These compounds, which incorporate 1,2,3-triazole as an amide isostere, have antiproliferative properties against androgen cells (LNCaP and PC-3).

A CuAAC-based synthesis of a triflorcas analogue by replacing the amide bond with a 1,2,3-triazole was described by Colombo et al. [118]. This replacement allowed for the good maintenance of triflorcas inhibitory activity, such as the diffusion of MDCK cells (epithelial cells) and in vitro tumorigenesis of H1437 (lung cancer). 

This clearly shows the bioisostery between 1,2,3-triazole and amide, and encourages the use of copper-catalyzed Huisgen cycloaddition in the synthesis of drug analogues containing an amide bond.

**Figure 27:**
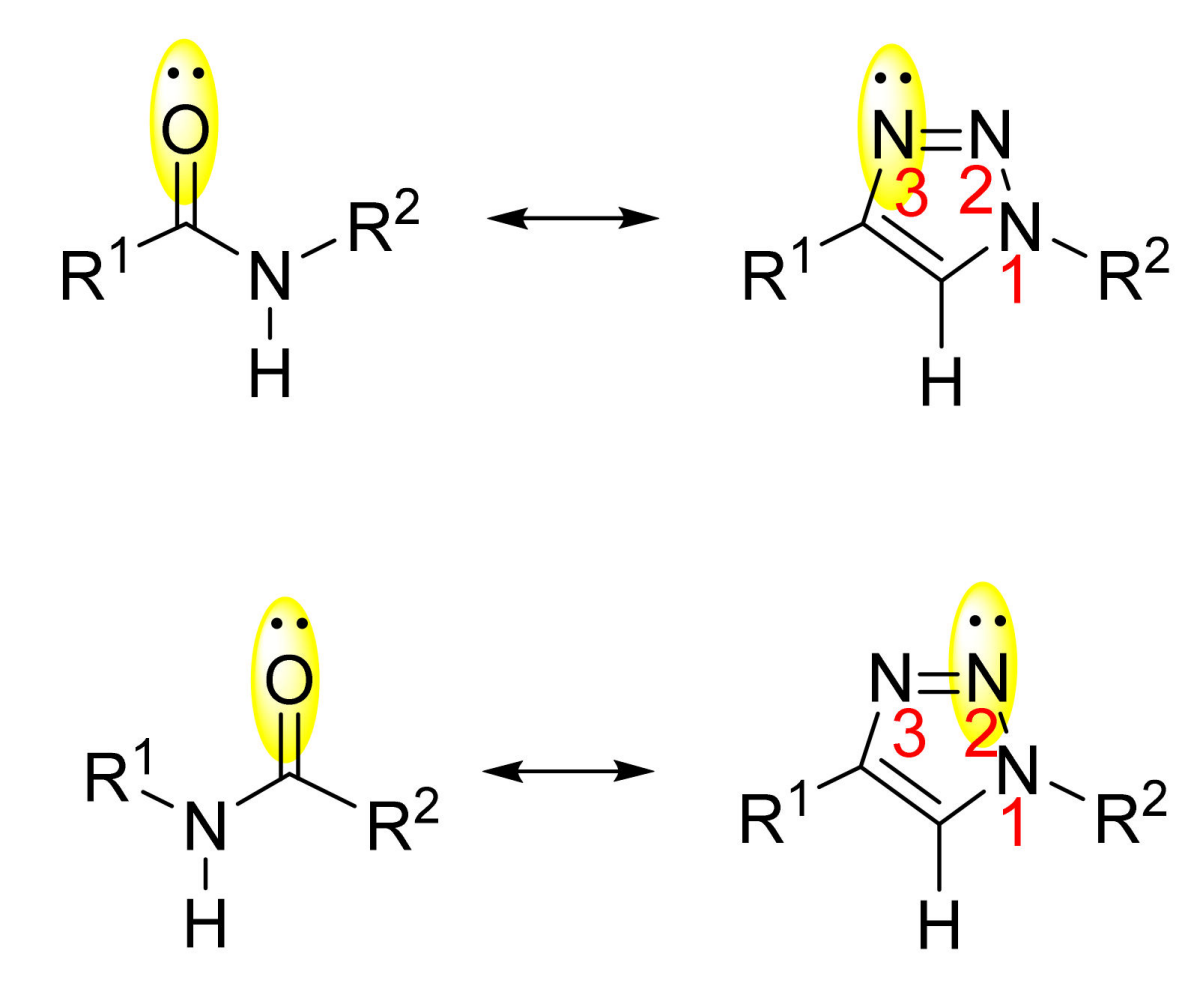
Different ways of mimicking amide bonds.

An important point to consider in drug synthesis and design is that triazoles can mimic amide bonds in different ways (Figure 27). In some cases, the lone pair of triazole N-2 mimics the carbonyl oxygen of the amide bond, instead of N-3, which is similar to the case of triazole derivatives of pantothenamides, where the arrangement of triazole is consistent with the similar IC_50_ values of compounds containing the amide bond and their triazole analogues [119,120].

The bioisosteric substitution with triazole moieties of the amide bond and the use of 1,2,3-triazole in the synthesis of peptoid hybrids proved advantageous. They enabled the synthesis of analogues with an affinity for receptors, proteolytic stability, and several improved properties. 

## 5. Conclusions

Since its initial development, the copper-catalyzed azide–alkyne cycloaddition reaction, CuAAC, has become a ligation strategy in synthetic organic chemistry. This reaction is used in peptide synthesis, because 1,2,3-triazoles closely resemble amide bonds while being stable to enzymatic degradation. These characteristics make 1,2,3-triazoles promising substrates as amide bond substitutes for the development of new peptidomimetics with potentially improved biological characteristics. Additionally, the triazolic peptide structure is flexible in terms of appropriately adjusting to the corresponding receptor or enzyme. 

This review has described the use of CuAAC in peptidomimetic synthesis while showing the similarity between 1,4-disubstituted 1,2,3-triazole and the peptide bond. It also discusses how CuAAC was used to insert triazoles into peptide chains, synthesize β-turn and multi-spiral α-helical secondary structures and triazolamer structures, and modify amino acid side chain and macrocycle peptidomimetics to improve biological activity and chemical and physical stability.

This review also shows how triazoles are used to link peptides to other functional moieties, such as carbohydrates, biotin, fluorescein, polymers, dendrimers, and metal complexes, and to prepare peptoid hybrids with improved properties.

Although CuAAC, the most important reaction in click chemistry, has been used in a variety of biomimetics in peptide science, we anticipate that it will continue to be used for the preparation and modification of novel triazole peptidomimetics due to the great interest in peptides as therapeutic drugs.
